# Maternal fecal microbiome predicts gestational age, birth weight and neonatal growth in rural Zimbabwe.

**DOI:** 10.1016/j.ebiom.2021.103421

**Published:** 2021-06-15

**Authors:** Ethan K. Gough, Thaddeus J. Edens, Hyun Min Geum, Iman Baharmand, Sandeep K. Gill, Ruairi C. Robertson, Kuda Mutasa, Robert Ntozini, Laura E Smith, Bernard Chasekwa, Florence D. Majo, Naume V. Tavengwa, Batsirai Mutasa, Freddy Francis, Lynnea Carr, Joice Tome, Rebecca J. Stoltzfus, Lawrence H. Moulton, Andrew J. Prendergast, Jean H. Humphrey, Amee R. Manges, SHINE Trial Team

**Affiliations:** 1 Department of International Health, Johns Hopkins Bloomberg School of Public Health, Baltimore, MD, USA; 2 Devil's Staircase Consulting, West Vancouver, British Columbia, Canada; 3 School of Population and Public Health, University of British Columbia, Vancouver, Canada; 4 Blizard Institute, Queen Mary University of London, London, UK; 5 Zvitambo Institute for Maternal and Child Health Research, Harare, Zimbabwe; 6 Department of Population Medicine and Diagnostics, Cornell University, Ithaca, NY, USA; 7 Department of Experimental Medicine, University of British Columbia, Canada; 8 Department of Microbiology and Immunology, University of British Columbia, Canada; 9 British Columbia Centre for Disease Control, Vancouver, British Columbia, Canada; 10 Goshen College, Goshen, Indiana, USA; 11 Members of the SHINE Trial team who are not named authors are listed in https://academic.oup.com/cid/article/61/suppl_7/S685/358186

**Keywords:** microbiome, metagenome, maternal, birth weight, pregnancy, gestation

## Abstract

**Background:**

Preterm birth and low birth weight (LBW) affect one in ten and one in seven livebirths, respectively, primarily in low-income and middle-income countries (LMIC) and are major predictors of poor child health outcomes. However, both have been recalcitrant to public health intervention. The maternal intestinal microbiome may undergo substantial changes during pregnancy and may influence fetal and neonatal health in LMIC populations.

**Methods:**

Within a subgroup of 207 mothers and infants enrolled in the SHINE trial in rural Zimbabwe, we performed shotgun metagenomics on 351 fecal specimens provided during pregnancy and at 1-month post-partum to investigate the relationship between the pregnancy gut microbiome and infant gestational age, birth weight, 1-month length-, and weight-for-age z-scores using extreme gradient boosting machines.

**Findings:**

Pregnancy gut microbiome taxa and metabolic functions predicted birth weight and WAZ at 1 month more accurately than gestational age and LAZ. *Blastoscystis sp, Brachyspira sp* and Treponeme carriage were high compared to Western populations. Resistant starch-degraders were important predictors of birth outcomes. Microbiome capacity for environmental sensing, vitamin B metabolism, and signalling predicted increased infant birth weight and neonatal growth; while functions involved in biofilm formation in response to nutrient starvation predicted reduced birth weight and growth.

**Interpretation:**

The pregnancy gut microbiome in rural Zimbabwe is characterized by resistant starch-degraders and may be an important metabolic target to improve birth weight.

**Funding:**

Bill and Melinda Gates Foundation, UK Department for International Development, Wellcome Trust, Swiss Agency for Development and Cooperation, US National Institutes of Health, and UNICEF.

**Research in context**

**Evidence before this study**

Preterm birth and low birth weight are a persistent public health challenge in low-income and middle-income countries (LMIC) that predict poor child health. There is need to identify more effective targets for intervention. A limited number of small studies, predominantly from high-income western populations, suggest that the maternal gut microbiome undergoes important metabolic changes during pregnancy that may play an underappreciated role in preterm, low birth weight and subsequent neonatal health. However, studies of the pregnancy gut microbiome in LMICs, where preterm and low birth weight are most prevalent, are extremely sparse; and metagenomic studies to date severely underrepresent populations outside of North America and Europe.

**Added value of this study**

We used shotgun metagenomics to investigate the relationship of pregnancy gut microbiome taxa and metabolic functions with infant gestational age, birth weight, and neonatal growth in a subsample of women and their infants enrolled in the Sanitation, Hygiene, Infant Nutrition Efficacy trial in rural Zimbabwe. In this population, resistant-starch degrading bacteria were the predominant gut taxa and were important predictors of birth weight and neonatal growth. Microbiome functions involved in signaling, starch, vitamin B and energy metabolism were also important predictors. Gut microbiome predictors of birth weight and neonatal growth largely reflected the low-diversity, resistant-starch dominated diet of the study population. This is the first study to identify gut microbiome markers of birth weight and early infant growth that likely reflect the dietary patterns of the mother, in a sub-Saharan African population where there is a very high prevalence of preterm birth and low birth weight.

**Implications of all the available evidence**

The pregnancy gut microbiome of rural Zimbabwean mothers, primarily the abundance of resistant-starch degraders, is an important contributor to birth weight and neonatal growth. Microbiome functions that predicted these outcomes suggested that bacterial capacity to respond to nutrient availability in the maternal diet may be important to promoting improved birth weight and early infant growth in populations with a monotonous starch-rich diet.

Alt-text: Unlabelled box

## Introduction

Children weighing <2,500 grams (g) at birth (low birth weight [LBW]), due to intrauterine growth retardation or preterm birth (<37 weeks completed gestation), are at high risk of morbidity and mortality during infancy and childhood, and of adverse effects over the life course [1]. Globally, LBW and preterm birth affect one in seven [2] and one in ten [3] livebirths respectively, primarily in low-income and middle-income countries (LMICs). However, nutritional interventions to promote optimal gestation, fetal and neonatal growth have not had a significant impact, or their effects have been considerably heterogeneous [4]. There is a need to better understand the underlying nutritional and environmental determinants of LBW and preterm birth so more effective targets for intervention can be identified.

Recent evidence suggests that the composition and function of the maternal gut microbiome before and during pregnancy may affect birth weight and duration of gestation [5], [6], [7], [8]. Changes in maternal gut microbiome composition [9], [10], [11] and metabolic activity [12] have been observed during pregnancy; for example, increased carbohydrate metabolism and butyrate production [12]. These alterations in the maternal microbiome may contribute directly to fetal growth, or indirectly through influences on maternal nutritional status and gestational weight gain. In contrast, other studies have reported no differences in gut microbiome composition during pregnancy [9] or have reported that the effect of other host characteristics such as pre-pregnancy weight, gestational weight gain, fasting blood glucose, and place of residence explain 3-5 fold more variance in microbiome composition than stage of pregnancy [13].

The gut microbiome can directly suppress [14,15] or promote [16] intestinal inflammation, and has been associated with biomarkers of environmental enteric dysfunction [17,18]. Biomarkers of intestinal damage and microbial translocation that indicate enteric dysfunction, in turn, have been associated with shorter gestation [19], [20], [21] and smaller birth size [21]. Enteric pathogens also promote intestinal inflammation and are associated with alterations in microbiome composition [22]. Inadequate sanitation and hygiene have been linked to preterm birth in LMICs [23] where exposure to enteric pathogens is prevalent [24].

Nutrition during pregnancy can also influence fetal growth and development [25,26]. Dietary influences on the gut microbiome are well established [27,28], and the intestinal microbiome plays an important role in nutrient harvesting [29]. The gut microbiome can regulate blood pressure through the production of short chain fatty acids (SCFAs) [30] from dietary fiber [31]. Production of SCFAs by the gut microbiome during pregnancy has been associated with lower blood pressure in observational studies [8] and pregnancy microbiome composition has been associated with pre-eclampsia [32], which is an important risk factor for preterm birth [33]. In addition, the gut microbiome may contribute to prevention of micronutrient deficiencies, such as folate deficiency [34,35]. Plasma folate has been related to LBW when deficient [36], and higher carriage of intestinal *Bifidobacteria sp* was positively associated with plasma folic acid [37].

Indirectly, the gut microbiome may affect pregnancy outcomes through an impact on maternal nutritional status and gestational weight gain during pregnancy, which influence fetal growth. In adults, gut bacteria-derived SCFAs regulate food intake, body weight, energy expenditure, and satiety [38,39], and oral probiotic administration has been shown to regulate central adiposity during and after pregnancy [40]. In observational studies, mothers who were overweight prior to pregnancy or who gained more weight during pregnancy showed greater gut *Bacteroides, Clostridium, Staphylococcus* and *Enterobacteriaceae* (*Escherichia coli*) abundance [37,41].

Furthermore, the maternal gut is a predominant source of bacteria to colonize the infant gastrointestinal tract at birth [42,43]. Childhood microbiome immaturity (delayed acquisition of specific taxa with child age) is related to early-life growth [44], [45], [46]. A dysbiotic maternal gut microbiome can, in part, be directly transferred to the newborn [47], regardless of whether the dysbiosis results from dietary deficiencies or pathogen exposure. Growth during the neonatal period may, therefore, also be impacted via acquisition of the maternal pregnancy gut microbiome by the infant at birth.

However, the evidence for a relationship between the pregnancy gut microbiome and adverse birth outcomes comes predominantly from high-income settings, and the results of these small studies have been inconsistent [48]. Moreover, human microbiome species from settings outside of high-income European and North American populations are severely underrepresented, which highlights the need for evidence from more diverse populations and environments [49]. We sought to better understand the role of the maternal microbiome during pregnancy on gestational age, birth weight, and neonatal growth. Our hypothesis is that pathogen carriage, and bacterial species and functions in the fecal microbiome of pregnant mothers that are related to diet, water, sanitation, and hygiene will predict gestational age, birth weight and neonatal growth. We investigated our hypothesis in a subsample of mothers and infants participating in the Sanitation Hygiene Infant Nutrition Efficacy (SHINE) Trial [50]. SHINE was a 2 × 2 factorial cluster-randomized trial designed to test the impact of improved household water quality, sanitation, and hygiene (WASH) and improved infant and young child feeding (IYCF) on linear growth and anaemia at 18 months (mo) of infant age in rural Zimbabwe [50].

We performed whole metagenome shotgun sequencing of maternal fecal specimens collected during pregnancy and at 1mo post-partum to identify maternal microbes and metabolic functions that might influence infant gestational age, size at birth, or growth during the neonatal period. We also investigated the impact of the SHINE WASH intervention, baseline sanitation and hygiene-related factors, and maternal characteristics on the maternal fecal microbiome.

## Methods

### Study design and participants

The study design and methods for the SHINE trial and for microbiome analyses have been reported previously [51,52]. In brief, SHINE was a cluster-randomized trial of the independent and combined effects of improved IYCF and WASH in two rural Zimbabwean districts with 15% antenatal HIV prevalence. The WASH intervention was designed to limit exposure to human and animal feces, was initiated during pregnancy, and included, at the household level: construction of a ventilated improved pit latrine, installation of two hand-washing stations plus monthly delivery of liquid soap and water chlorination solution, provision of a play space for the infant, and hygiene counseling. Infants had birth weight measured in institutions by personnel who had been provided with Seca scales and had been trained in their use. At 1mo of infant age, a home visit was conducted to measure weight and length, as previously described [50]. The SHINE trial is registered at ClinicalTrials.gov (NCT01824940).

In a pre-specified substudy of SHINE, 1,656 mother-child pairs were selected for biological specimen collection from the mother during pregnancy then at 1mo post-partum, and intensive collection from the infant at 1, 3, 6, 12 and 18 months of follow-up. This substudy sample was enriched for HIV-positive mothers because we were also interested in investigating outcomes associated with fetal HIV-exposure. Of these substudy pairs, two hundred seven mother-child pairs with nearly complete longitudinal fecal sample collection were investigated as part of the fecal microbiome study; these are the focus of our analyses in this manuscript.

### Fecal specimens

Mothers collected fecal specimens prior to a home visit by a research nurse during pregnancy and at the 1mo post-partum study visit. Fecal specimens were immediately placed in a cold box and transported to the regional laboratory, where they were stored at -80°C until transfer to the central laboratory in Harare for long-term archiving at -80°C. Fecal specimens were transferred via private courier on dry ice from Harare, Zimbabwe to Vancouver, British Columbia. The Qiagen DNeasy PowerSoil Kit was used to extract total DNA from 200mg of feces, according to manufacturer's instructions.

### Whole metagenome library preparation and sequencing

Paired-end libraries were constructed using the Illumina TruSeq kit and using New England Biosystem TruSeq compatible library preparation reagents. Libraries were sequenced at the British Columbia Genome Sciences Centre using the Illumina HiSeq 2500 platform. Forty-eight libraries were pooled and included per sequencing lane. Sixteen negative process controls, consisting of sterile water subjected to DNA extraction, library preparation, and sequencing, were included to capture microbial contamination of laboratory reagents.

### Bioinformatics

Sequenced reads were trimmed of adapters and filtered to remove low-quality, short (<60 base-pairs), and duplicate reads, as well as those of human, other animal or plant origin using KneadData with default settings [53]. Species composition was determined by identifying clade-specific markers from reads using MetaPhlAn3 with default settings [54]. Relative abundance estimates were obtained from known assigned reads, and unknown read proportions were estimated from total, assigned and unassigned, reads. Percent human DNA was estimated from KneadData output, using the proportion of quality-filtered reads that align to the human genome. DNA extraction from fecal samples did not include enrichment for viral DNA, nor was cDNA synthesized. Given the smaller viral genome sizes, sequencing depth, and limitations of MetaPhlAn3 for virus identification, we did not include viruses in our current analyses. We applied a minimum relative abundance threshold of 0.1% for taxa and included taxa meeting this threshold in ≥5% of specimens in all downstream analyses.

Functional gene and metabolic pathway composition was determined using HUMAnN3 with default settings against the UniRef90 database [55]. Functionally annotated reads were further classified into level-4 enzyme commission (EC) categories using provided scripts. Enzyme family and pathway abundance estimates were normalized using reads per kilobase per million mapped reads (RPKM) and then re-normalized to relative abundance. We applied a minimum relative abundance threshold of 3 × 10^−7^% for EC and pathway features, at ≥5% prevalence, in all downstream analyses.

### Statistical analyses

All analyses were conducted in R version 3.3.2 [56]. Infant weight and length at 1mo of age were converted to Z-scores using the WHO growth standard [57]. The association between epidemiologic variables and birth weight or infant growth was assessed using multivariable linear regression. Epidemiologic variables associated with infant growth were selected for inclusion in XGBoost models.

Maternal fecal microbiome composition was investigated using descriptive approaches (e.g. boxplots) and α- and β-diversity metrics. α-diversity was assessed using the Shannon, Simpson, inverse Simpson, evenness and richness metrics; while β-diversity was evaluated using the Bray–Curtis dissimilarity index and visualized using principal coordinate analyses (PCoA) performed in the package *vegan* [58]. Permanova was performed to assess significant differences in β-diversity by visit, HIV-status, season of birth, and WASH assignment using the *adonis* function [59] in *vegan*.

The bivariate relationship between epidemiologic variables and maternal fecal taxa was examined using zero-inflated beta regression fitted by generalized additive models for location, scale and shape (GAMLSS), with a log-link, in the R package *gamlss* [60]. A separate model was fit for each microbiome taxon, yielding a relative abundance ratio (RAR). Epidemiologic variables were assessed individually and included baseline household characteristics (number of occupants, diet diversity, food insecurity, wealth index, improved floor, improved latrine, time to water, water treatment), maternal characteristics (age, height, mid-upper-arm circumference (MUAC), education, religion, HIV-status, anti-retroviral therapy, depression score, parity), mode and location of delivery, season of fecal specimen collection, diet composition on a normal day, randomized WASH allocation, and percent human DNA. To assess diet composition on a normal day, mothers responded to questions about her diet and her family's diet (meals and snacks) consumed ‘yesterday’, unless yesterday was a feast day or celebratory day, then she was asked to respond for the day before or her last ‘normal’ diet day. False discovery rate (FDR) adjustment was used to calculate q-values from p-values [61]. Results are reported when the q-value was less than 0.05.

Independent associations between the maternal pregnancy microbiome and gestational age, birth weight, WAZ or LAZ at 1mo was evaluated using extreme gradient boosting machines (XGBoost). Microbiome data are very complex, consisting of hundreds of species or pathways, and thousands of enzyme commissions (EC), and comprise of highly right-skewed distributions with many zeros. Analytic methods that can address these challenges and select the most important subset of features for a given outcome are limited. XGBoost builds an optimized predictive model by creating an ensemble from a series of weakly predictive models. In aggregate, each additional model that is fitted is parameterized to improve the overall prediction accuracy of the ensemble when the new model is combined with previous models. Only features that improve model accuracy are retained in the process. XGBoost is also non-parametric, can capture non-linear relationships, and can accommodate high-dimensional data [62]. XGBoost or other decision-tree based methods (eg. Random Forests) have been applied in other microbiome analyses [45,63,64].

The XGBoost models were developed using microbiome relative abundances, diversity measures, percent human DNA reads, percent unknown reads, and selected epidemiologic variables. Epidemiologic variables were selected for inclusion in the XGBoost model based on: (i) their association with birth weight, LAZ or WAZ at 1mo in multiple linear regression models (Supplemental Table 4); (ii) their association with microbiome diversity indices in simple linear regression models (p<0.05); and (ii) their association with microbiome taxon abundance in GAMLSS models (q<0.05). XGBoost model selection was performed in 3 stages. In stage one, the *BayesianOptimization* function of the *rBayesianOptimization* package was used with 10-fold cross-validation to select model hyperparameters (Supplementary Table 1) by minimizing the mean squared error (MSE). Models with the lowest MSE (in the 5^th^ percentile) were retained, and from these models the variables that contributed to the top 95% of variable importance by proportion were retained. In stage two, all epidemiologic variables were included with the microbiome variables obtained in stage one. *BayesianOptimization* was run as described in stage one but using leave-one-out cross-validation. Microbiome variables that contributed to the top 95% of variable importance by proportion were retained. In stage three, all epidemiologic variables, microbiome features, and hyperparameters selected in stage two were used to fit our final models. Final models were fit using leave-one-out cross-validation to minimize the MSE. This process was implemented separately for each outcome (gestational age, birth weight and neonatal growth measures), and separately for microbiome taxa, pathways, and enzymes. Finally, all models were re-run excluding epidemiological variables to assess the contribution of the maternal pregnancy microbiome alone to the accuracy of our final models. XGBoost models were fitted using the H20.ai engine and *h2o* R package interface with the *XGBoost* package.

XGBoost model performance was evaluated using three estimates: (i) pseudo-R-squared (pseudo-R^2^) between the final XGBoost-predicted outcome value and the observed outcome value, where pseudo-R^2^=1·0 is perfect prediction; (ii) the mean absolute error (MAE), which is defined as the average absolute difference between observed and predicted outcome values estimated from cross-validation and summarizes model performance in the actual units of the outcome variable (kilograms for birth weight, weeks for gestational age, standard deviations for LAZ and WAZ at 1mo); and (iii) the root mean squared error, which is defined as the average squared difference between observed and predicted outcome values estimated from cross-validation. We used the scaled relative importance for each variable in a model to identify the twenty most informative variables for further interpretation, where the most important variable is ranked first, and the importance of subsequent variables are relative to the first variable.

The marginal relationships between the twenty most important epidemiologic variables, microbiome species, pathway or enzyme relative abundances and each outcome were visualized for interpretation [65] using accumulated local effects plots (ALE). ALE plots can be interpreted as showing a marginal effect, adjusted for all covariates retained in the final model. That is, the plots show the expected change in the outcome variable per increment in a feature, either epidemiologic or microbiome, adjusted for the variables retained in the model. The range of the feature across all observations is partitioned into intervals such that each interval contains roughly the same number of observations. The corresponding expected changes in the outcome values per increment, or effect sizes, are averaged. The resulting effect sizes are plotted cumulatively and centered about the average effect size [66]. ALEs were generated using the *ALEplot* package and were plotted using *ggplot2*. Standard deviations were calculated per increment and were used to calculate and plot increment-wise 95% confidence intervals.

### Ethics statement

All SHINE mothers provided written informed consent. The Medical Research Council of Zimbabwe (MRCZ/A/1675), Johns Hopkins Bloomberg School of Public Health (JHU IRB # 4205.), and the University of British Columbia (H15-03074) approved the study protocol, including the microbiome analyses.

### Data sharing

All relevant data are within the paper and its Supporting Information files except for the raw data which the trial team will begin loading as individual participant data with an accompanying data dictionary at http://ClinEpiDB.org in mid-2021. Prior to that time, the data are housed on the ClinEpiDB platform at the Zvitambo Institute for Maternal and Child Health Research and available upon request from Ms. Virginia Sauramba (vsauramba@zvitambo.co.zw).

### Role of funding source

The funders had no role in study design, data collection, data analyses, interpretation, or writing of the manuscript.

## Results

### Study population

**Figure 1:**
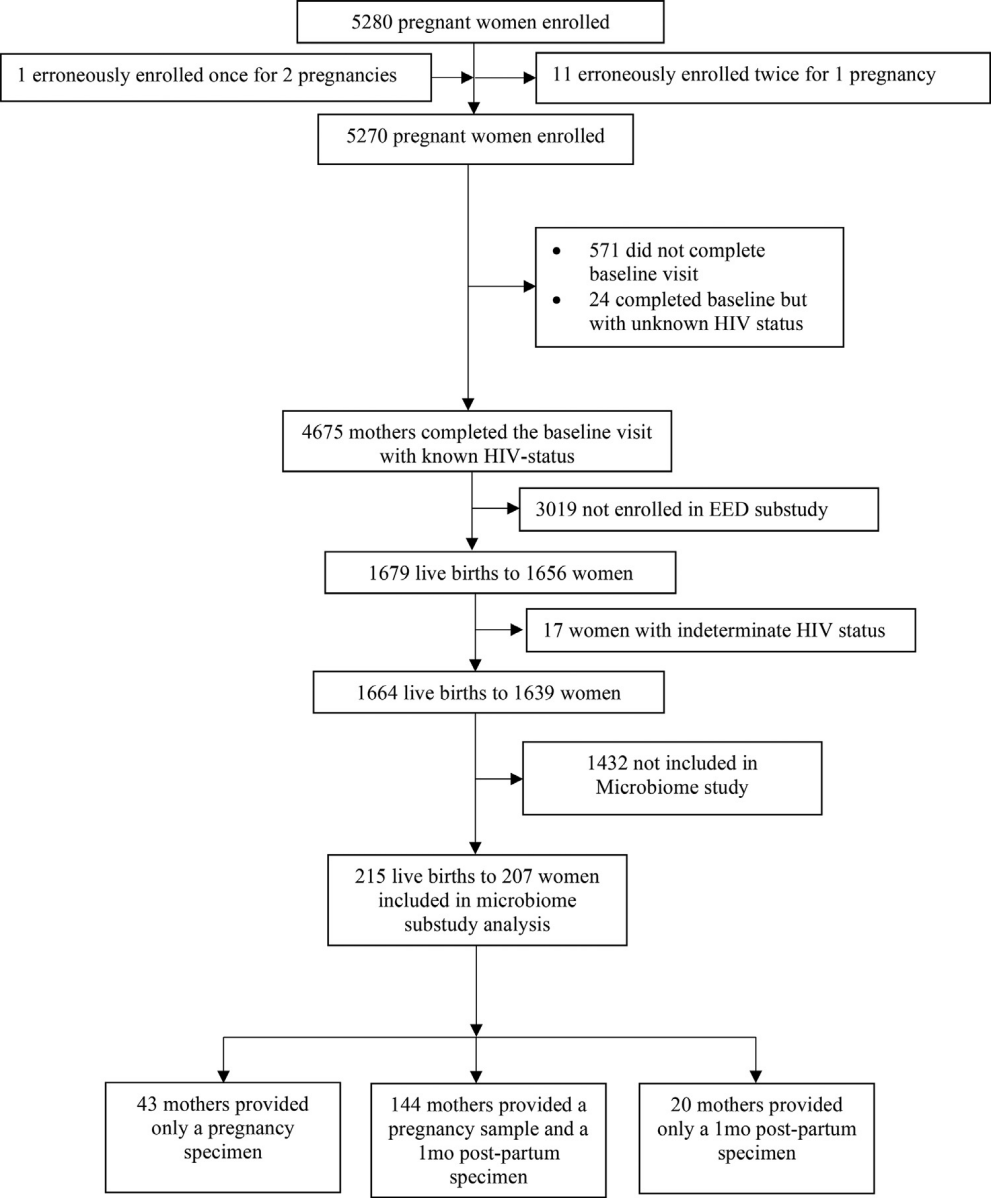
**Flow of participants through the SHINE microbiome substudy**.

Two hundred seven SHINE mothers were included in these analyses (Figure 1). These mothers gave birth to 215 infants (199 singletons and 8 sets of twins). A comparison of mothers included in the microbiome substudy and the entire SHINE cohort is provided in Table 1. There were 97 mothers living with HIV in the substudy, with median (IQR) CD4 count of 444 cells/mm^3^ (300,652). The majority (93%) were receiving antiretroviral therapy (ART) and 46% were receiving cotrimoxazole prophylaxis at baseline (Table 1). In the microbiome substudy compared to the overall SHINE cohort, there were slightly fewer mothers allocated to the WASH intervention arm and fewer mothers in the lowest wealth quintile, more HIV-positive mothers with a handwashing station, and more HIV-negative mothers with livestock in the home at baseline. Approximately 45% of households met minimum dietary diversity standards, and few households experienced severe food insecurity (Table 1). Mothers and infants included in these analyses otherwise resembled the overall SHINE population (Table 1). Importantly, 100% of mothers consumed cereals on a typical day (Supplementary Table 2). In Zimbabwe, the most commonly consumed cereal is maize, a major source of resistant-starch.

**Table 1:** Baseline characteristics of the microbiome sub-study versus all SHINE mothers and their infants

Characteristic	SHINE mothers included in the microbiome sub-study n = 207		All SHINE mothers completing the baseline visit n = 4,675	
	HIV-positive n = 97	HIV-negative n = 110	HIV-positive n = 773	HIV-negative n = 3902

Trial Arm				
Standard of care	23 (23.7%)	32 (29.1%)	173 (22.4%)	911 (23.4%)
IYCF	28 (28.9%)	32 (29.9%)	164 (21.2%)	929 (23.8%)
WASH	20 (20.6%)	20 (18.2%)	214 (27.7%)	996 (25.5%)
WASH + IYCF	26 (26.8%)	26 (23.4%)	222 (28.7%)	1066 (27.3%)
Household Characteristics				
Median Number of Occupants (IQR)	4.0 (3.0,5.0)	5.0 (3.0,6.0)	4.0 (3.0, 6.0)	5.0 (3.0, 6.0)
Wealth Quintile				
1 (lowest)	18 (20.0%)	9 (9.3%)	210 (27.5%)	714 (18.5%)
2	21 (23.3%)	24 (24.7%)	178 (23.3%)	752 (19.5%)
3	21 (23.3%)	19 (19.6%)	150 (19.6%)	775 (20.1%)
4	17 (18.9%)	26 (26.8%)	112 (14.6%)	823 (21.3%)
5 (highest)	12 (13.3%)	19 (19.6%)	115 (15.0%)	801 (20.7%)
Sanitation				
Improved latrine at household	29 (32.2%)	36 (37.1%)	217 (28.7%)	1227 (32.2%)
Water				
Main source of household drinking water improved	63 (70.0%)	60 (61.9%)	450 (59.6%)	2440 (63.7%)
Median one-way walk time to fetch water (IQR), min	10 (5,15)	10 (5,15)	10.0 (5, 20)	10.0 (5, 20)
Median one-way walk time to fetch water (IQR), min	10 (5,15)	10 (5,15)	10.0 (5.0, 20.0)	10.0 (5.0, 20.0)
Treat drinking water to make it safer	15 (15.9%)	15 (14.2%)	84 (12.1%)	449 (12.6%)
Hygiene				
Improved floor at household	44 (47.7%)	69 (62.7%)	361 (47.8%)	2118 (55.6%)
Maternal characteristics				
Mean age (sd), years	31.2 (5.9)	27.2 (6.7)	29.0 (6.3)	25.7 (6.7)
Mean height (sd), cm	161.0 (6.2)	160.6 (5.4)	159.6 (9.0)	159.8 (8.1)
Mean mid-upper-arm circumference (sd), cm	27.0 (2.7)	27.3 (3.5)	26.3 (3.0)	26.4 (3.1)
Mean years of schooling completed (sd)	9.2 (2.2)	9.4 (1.9)	9.2 (2.1)	9.6 (1.8)
Median parity (IQR)	2.0 (1.0,3.0)	2.0 (1.0,3.0)	2.0 (1.0, 3.0)	2.0 (1.0, 3.0)
Married	85 (94.4%)	89 (91.8%)	691 (94,5%)	3550 (95.8%)
Religion				
Apostolic	50 (55.6%)	30 (31.0%)	348 (47.2%)	1732 (46.4%)
Other Christian	35 (38.9%)	63 (64.9%)	313 (42.4%)	1699 (45.5%)
Other	3 (3.3%)	2 (2.1%)	77 (10.4%)	306 (8.2%)
HIV status and therapy				
Mean CD4 count (sd)	444 (300,652)	NA	460.5 (349, 614)	NA
Currently receiving cotrimoxazole	32 (45.7%)	NA	399 (55.7%)	NA
Currently receiving ART	65 (92.6%)	NA	582 (81.2%)	NA
Potential depression (≥ 10 on Edinburgh Postnatal Depression Scale	11 (11.7%)	6 (6%)	103 (14.0%)	350 (9.4%)
Trimester of Specimen collection				
First trimester (0 to ≤84 weeks)	13 (15.1%)	21 (22.8%)		
Second trimester (>84 to ≤196 weeks)	65 (75.6%)	56 (60.9%)		
Third trimester (>196 weeks)	8 (9.3%)	15 (16.3%)		
Diet quality and food security				
Household meets minimum Diet Diversity Score	35 (43%)	44 (44%)	252 (38.2%)	1364 (40.2%)
Median Coping Strategies Index score (IQR)	0 (0,3)	1 (0,4)	2.0 (0, 10)	1.0 (0, 7)
Mean days of staple food in the household (sd)	144 (119)	176 (120)	114.6 (146)	129.3 (129)
Season of stool collection				
Dry (May to September)	40 (44.4%)	59 (57%)	281 (39.4%)	1566 (42.5%)
Rainy (October to April)	50 (55.6%)	44 (43%)	433 (60.6%)	2120 (57.5%)
Infant Characteristics*				
Female sex	48 (48%)	50 (43%)	363 (50.1%)	1830 (49.1%)
Mean birth weight (sd), kg	3.0 (0.5)	3.0 (0.5)	3.0 (0.5)	3.1 (0.5)
Birthweight < 2500 g	13 (13%)	11 (10%)	84 (11.5%)	306 (9.2%)
Preterm (<37 weeks gestation)	6 (9.8%)	9 (11.5%)	63 (17.3%)	326 (16.3%)
Vaginal delivery	91 (96%)	104 (94%)	63 (17.3%)	326 (16.3%)
Institutional delivery	81 (86%)	107 (96%)	612 (92.5%)	3207 (92.4%)
Exclusive breastfeeding initiation	89 (98%)	103 (95%)	545 (83.6%)	3026 (88.7%)
Mean LAZ at 1-month (sd)	-1.1 (1.3)	-0.9 (1.3)	-1.1 (1.3)	-0.9 (1.4)
Mean WAZ at 1-month (sd)	-0.6 (1.4)	-0.3 (1.5)	-0.8 (1.3)	-0.5 (1.2)

* *n=215 infants born to 207 mothers (100 born to HIV-positive mothers and 115 born to HIV-negative mothers). Accurate gestational age measurement was unavailable for 20 HIV-exposed infants and 13 HIV-unexposed infants; and twins were excluded from gestational age estimation. n, sample size; IYCF; infant and young child feeding; WASH, water sanitation, and hygiene; IQR; inter-quartile range; min, minutes; sd; standard deviation; cm; centimeters; ART, antiretroviral therapy; Kg, kilograms; g, grams; AGA, adequate size for gestational age; SGA small for gestational age; LAZ, length-for-age z-score, WAZ, weight-for-age z-score.

A total of 351 whole metagenome sequencing datasets were produced. 144 mothers provided samples from the gestational visit (between 9- and 37-weeks gestation) and at 1mo post-partum (between 12 and 90 days after birth); an additional 43 provided only a pregnancy sample and 20 only a postpartum sample. There were median 1(IQR: 1,2) and 2(IQR: 1,4) datasets per randomization cluster at each visit, respectively. Since there was little opportunity for expression of intra-cluster correlation, it was not adjusted for in any of our analyses. There was very little variability in sequencing depth across specimens (mean 11·4±3·0 million quality-filtered read pairs for all datasets). Negative controls revealed negligible levels of microbial contamination of reagents (mean 734±3,462 quality-filtered reads) relative to the sequencing depth achieved across specimens. The median proportion of human reads detected was 0·01% but ranged widely from 0·0004% to 19·3% (Supplementary Figure 1), indicating high human DNA enrichment in some fecal specimens.

After applying our relative abundance and prevalence threshold criteria, 144 unique taxa at the species level or above, 424 microbiome pathways, and 1,956 enzyme commission features were included in downstream analyses from the pregnancy and 1mo post-partum metagenome datasets. Of these, 39 taxa (28%) were defined by MetaPhlAn3 as co-abundance gene groups (CAGs) [67]. CAGs are putative species genomes derived from assembled sequences in publicly available metagenomes, and for which no bacterial culture-derived representative exists [49,67]. These represent novel bacteria that are prevalent in rural Zimbabwean mothers and that are missing from reference databases comprised of bacteria isolated or sequenced primarily from North American and European populations. Eight of these CAGs were defined at the phylum (Firmicutes) level only, meaning no genetically closer relative exists in reference databases at finer taxonomic levels.

### Baseline variables influencing maternal microbiome composition during pregnancy and 1mo post-partum

In PCoA analyses of microbiome β-diversity by epidemiologic variables (using Bray-Curtis distances), maternal microbiome composition differed modestly by HIV-status (Permanova p-value = 0·012, goodness of fit=0·19) and randomized WASH assignment (p-value = 0·022, goodness of fit = 0·23) (Supplementary Figure 2). However, microbiome composition did not vary by trimester of pregnancy (p-value = 0.343) (Supplemental Figure 3).

In bivariate analyses of differences in relative abundance of individual taxa by epidemiologic variables using GAMLSS models, only one taxon differed by maternal HIV-status and two taxa differed by WASH intervention, after FDR adjustment, at the post-partum visit only. Higher *Treponema berlinense* (RAR=4·67, q=0·003) was observed in HIV-negative mothers at the post-partum visit; while higher *Brachyspira sp* CAG 700 (RAR = 6·9, q<0·001) and lower *Akkermansia muciniphila* (RAR=0·37, q=0·042) were observed in mothers assigned to the WASH intervention (Supplementary Table 3) at the post-partum visit. There was no evidence that any of the α-diversity metrics varied by these variables.

### Pathogen detection

Overall, based on MetaPhlAn3 results, carriage of pathogens was uncommon. At a minimum relative abundance of 0·1%, *Brachyspira pilosicoli*, a spirochete associated with human zoonotic spirochaetosis, was carried by 20 mothers, while 22 mothers carried *Brachyspira sp* CAG 700. Interestingly, lower wealth quintile, lower primary education and greater maternal height were associated with higher *Brachyspira sp* abundance (Supplementary Table 3). Higher percentage of human DNA was associated with higher relative abundance of *Blastocystis* and *Alistipes shahii* at 1mo postpartum (Supplemental Table 3). Other non-pathogenic spirochetes were identified in many SHINE mothers, including *Tremponema succinifaciens* (n=68) and *Treponema berlinense* (n=17) (Supplementary Figure 4). Spirochetes have been shown to be over-represented in many non-Western populations [68]. Thirty-one mothers were also positive for *Blastocystis sp* subtype 1 during at least one visit. *Giardia intestinalis* or *Cryptosporidium spp* were detected but below the 0·1% relative abundance threshold. *Shigella* was not detected and *Salmonella enterica, Campylobacter jejuni* and *C. coli* were also present at <0·1% relative abundance. *Escherichia coli* was common, but MetaPhlAn3 does not separate by *E. coli* pathotype; therefore, presence of enteroaggregative or enteropathogenic *E. coli* could not be assessed.

### Epidemiologic variables, birth weight, and neonatal growth

In multivariable linear regression models, maternal height, MUAC, and gestational age, were associated with infant birth weight, as expected (Supplementary Table 4). While maternal MUAC and gestational age were associated with WAZ and LAZ at 1mo respectively.

### Maternal microbiome diversity, birth weight, and neonatal growth

To ensure temporality between maternal microbiome characteristics and pregnancy outcomes, all analyses of the association between maternal microbiome, gestational age, birth weight, and neonatal growth utilized the pregnancy visit metagenome datasets only. In simple linear regression models, microbiome Shannon diversity was not associated with infant birth weight or LAZ at 1mo (Supplementary Figure 5) but was associated with higher WAZ at 1mo (Supplementary Figure 5). Higher pregnancy microbiome taxon evenness was associated with higher infant WAZ at 1mo, and there was evidence for a positive association with the Shannon diversity index (*p*-value = 0·09).

### XGBoost model performance

Variables chosen for inclusion in XGBoost model building were infant sex, HIV-status, maternal MUAC, maternal height, birth season, pregnancy trimester and season of maternal specimen collection, microbiome diversity metrics, percent unknown, percent human DNA, and gestational age (for growth models). Performance measures for final models of microbiome species, ECs, or pathways, including or excluding epidemiologic variables, are presented in Supplementary Table 5. For all outcomes, the performance of models that included only microbiome features performed as well as the models that also included epidemiologic variables, indicating that model performance was predominantly driven by microbiome composition. Overall, model performance was best for birth weight, followed by neonatal growth, then gestational age. In terms of microbiome features, pathway relative abundance was more predictive than species abundance, while EC abundance was as good or better than pathway abundance at predicting outcomes, although this may be driven by the higher overall number of ECs.

### Important XGBoost variables for birth weight

**Figure 2:**
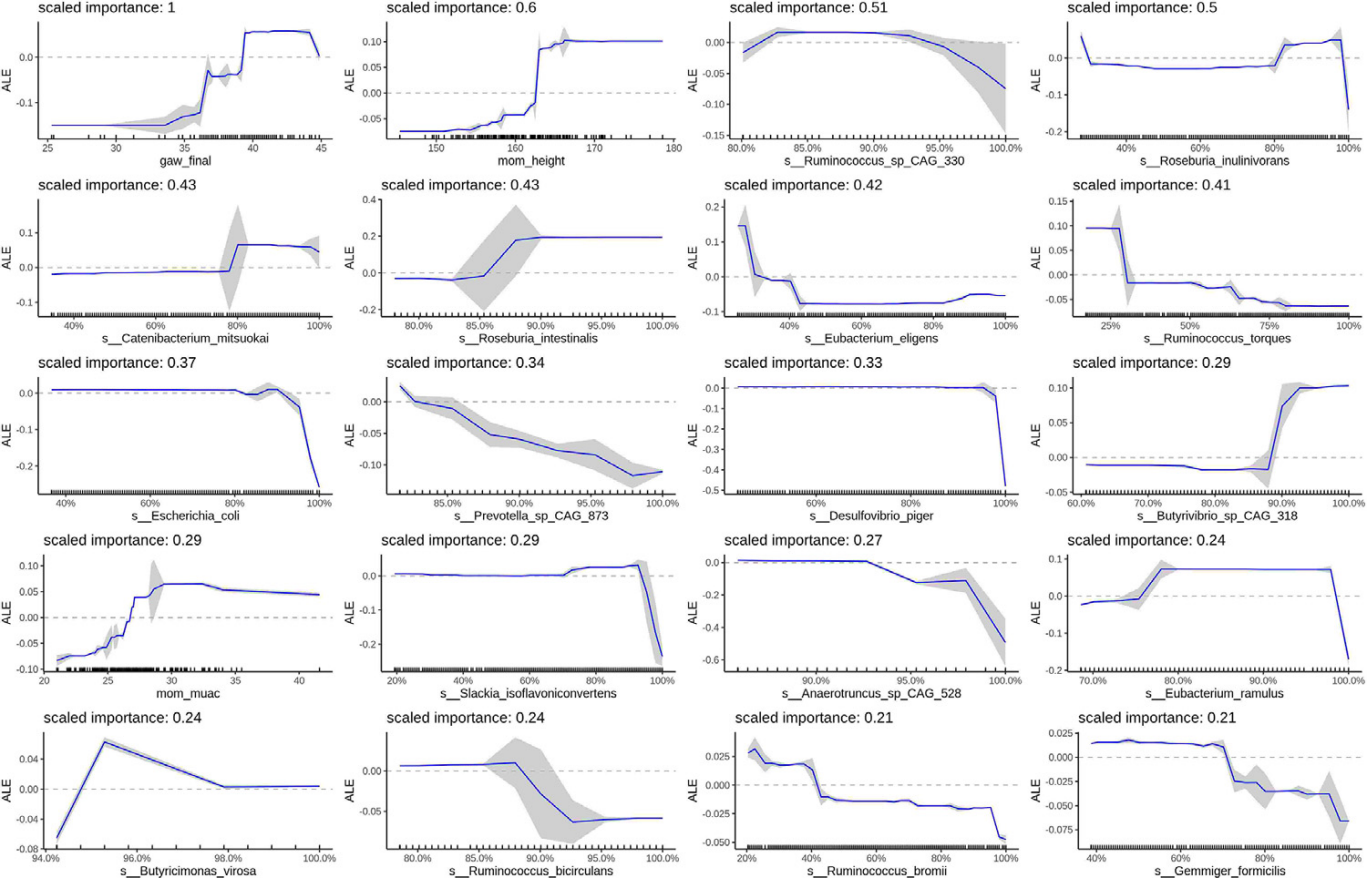
**Relationships between infant birth weight in Kg, epidemiologic variables, and maternal gut microbiome species relative abundance.** The top 20 predictors of infant birth weight by variable importance score are shown. For microbiome abundances, the x-axis represents the percentile of the abundance distribution. Epidemiologic and microbiome diversity variables are on the original scale. Tick marks on the x-axis are a rug plot of individual feature abundance percentiles. ALEs were generated using the *ALEplot* package and were plotted using *ggplot2*. Standard deviations (sd) were calculated per increment in microbiome feature and were used to calculate and plot increment-wise 95% confidence intervals as the average change in the outcome ±1.96(sd/sqrt(n)), where n is the number of observed feature values, and sd is the standard deviation of the change in the outcome variable in an interval. gaw_final, gestational age; mom_height, maternal height in centimeters; mom_muac, maternal mid-upper arm circumference in millimeters; pct_human, percent human reads; pct_unknown, percent unknown reads.

**Figure 3:**
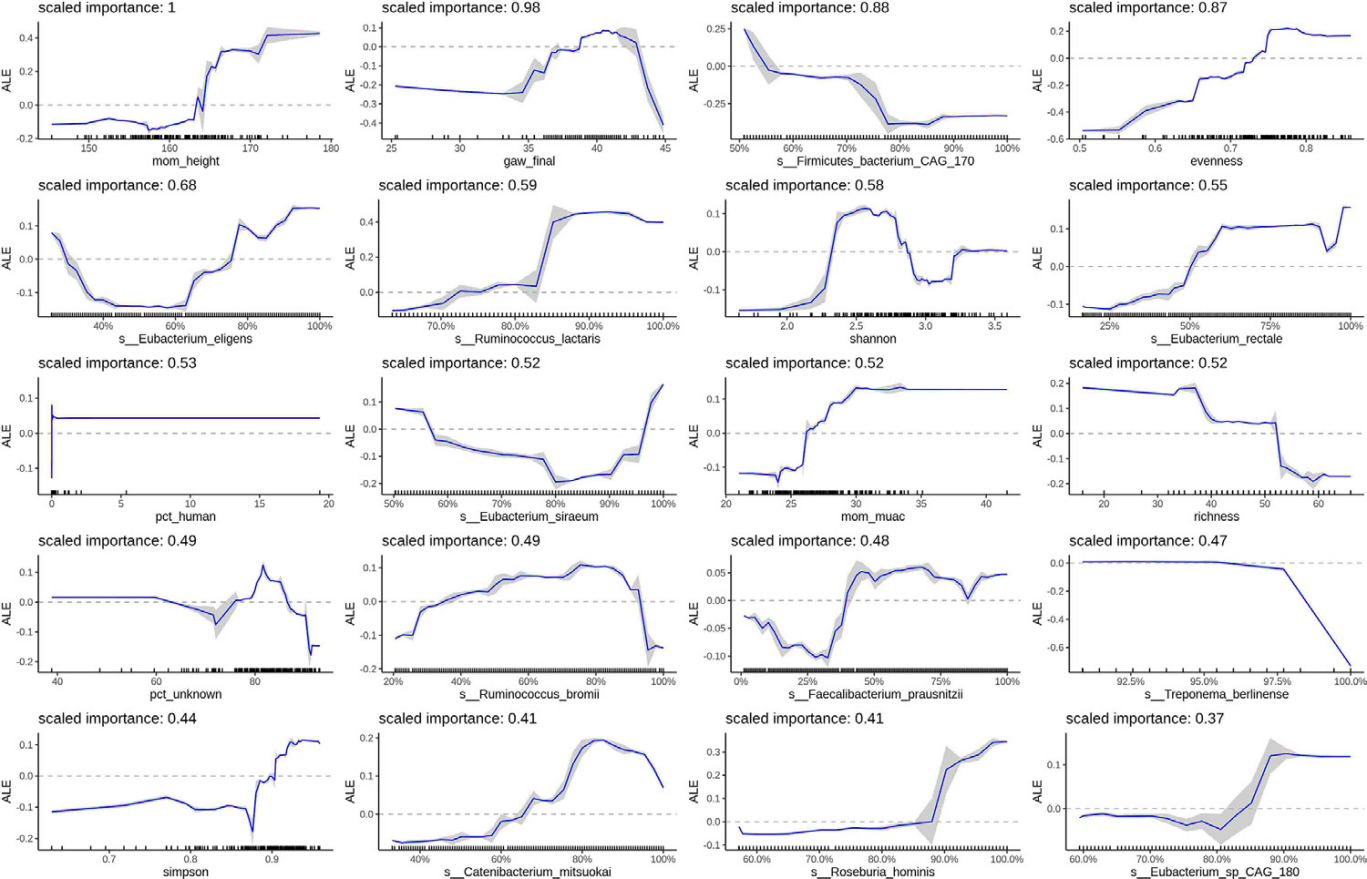
**Relationships between infant WAZ at 1-month, epidemiologic variables, and maternal gut microbiome species relative abundance.** The top 20 predictors of infant WAZ at 1-month by variable importance score are shown. For microbiome abundances, the x-axis represents the percentile of the abundance distribution. Epidemiologic and microbiome diversity variables are on the original scale. Tick marks on the x-axis are a rug plot of individual feature abundance percentiles. ALEs were generated using the *ALEplot* package and were plotted using *ggplot2*. Standard deviations (sd) were calculated per increment in microbiome feature and were used to calculate and plot increment-wise 95% confidence intervals as the average change in the outcome ±1.96(sd/sqrt(n)), where n is the number of observed feature values, and sd is the standard deviation of the change in the outcome variable in an interval. gaw_final, gestational age; mom_height, maternal height in centimeters; mom_muac, maternal mid-upper arm circumference in millimeters; pct_human, percent human reads; pct_unknown, percent unknown reads.

ALE plots are shown in Figure 2, Figure 3, and Supplementary Figures 6-15 to visually interpret the marginal relationships between each outcome and the individual top 20 variables in the final XGBoost models adjusted for the other epidemiologic variables and microbiome features retained in the model. Microbiome features are plotted using percentiles of the abundance distribution to improve visualization near zero where abundance data are denser. Epidemiologic and microbiome diversity variables are plotted on the original scale.

For infant birth weight, gestational age and maternal height were the most informative variables, predicting a cumulative increase in birth weight of 150g and 200g, respectively, over the range of observed values (Figure 2). Taxa that are associated with resistant-starch degradation, specifically members of the *Ruminococcaceae, Lachnospiraceae*, and *Eubacteriaceae* families [69,70], were the next most important predictors of birth weight. However, the relationships were quite variable, and most effects were only observed in the extremes of the abundance distributions. For example, *E. eligens* predicted a cumulative decrease of 250g from the lowest abundance up to approximately the 40^th^ abundance percentile, while *R. torques* predicted a cumulative decrease of 150g above the 25^th^ abundance percentile. In contrast, *R. intestinalis* and *Butyrivibrio sp CAG 318* predicted a cumulative increase of 200g and 100g, respectively, above the 80^th^ abundance percentile (Figure 2). Maternal MUAC was also among the 20 most important variables, predicting a 75g increase in birth weight.

The bacterial enzyme with the highest-ranking importance was glycogen synthase (~200g cumulative decrease up to the median abundance), exhibiting greater importance in the model than gestational age and maternal height (Supplementary Figure 6). Glycogen synthase is involved in starch metabolism. The remaining top 20 enzymes are involved in metabolic pathways that engage in signalling, energy, and vitamin B metabolism (Supplementary Figure 6). For example, histidine kinase (involved in environmental sensing) predicted a 300g increase in birth weight in the lowest abundance percentile. While thiazole biosynthesis pathway (vitamin B1 metabolism) predicted a 200g increase in the lowest abundance percentile and N10-formyl-tetrahydrofolate biosynthesis (vitamin B9 metabolism) predicted a 75g increased (Supplementary Figure 7).

Similarly, top ranking metabolic pathways are engaged in starch degradation, energy metabolism and signalling processes (Supplementary Figure 7). For example, sucrose degradation II pathway and the superpathway of anaerobic sucrose degradation predicted a 200g increase above the 80^th^ abundance percentile (Supplementary Figure 7). The signalling ppGpp biosynthesis pathway predicted an 85g decrease; while, in contrast, the signalling pathway purine ribonuceosides degradation predicted a ~150g increase (Supplementary Figure 7).

### Important XGBoost variables for WAZ at 1mo

For WAZ at 1mo, the most important taxa were also predominantly starch-degrading species in the families *Ruminococcaceae, Lachnospiraceae*, and *Eubacteriaceae* (Figure 3). One species outside of these families (*Catenibacterium mitsuokai*), that is also associated with fiber fermentation [71] and consumption of animal fat [72], predicted a ~0.3 sd increase in WAZ. *Treponema berlinense* predicted a 0.6 sd decrease above the 97^th^ abundance percentile. The Simpson diversity index and evenness were also important predictors, indicating a 0.2 sd and 0.7 sd increase through the range of observed diversity values, respectively. However, species richness predicted a 1 sd cumulative decrease in WAZ. As with birth weight, gestational age and maternal anthropometry were also important predictors of 1mo WAZ (Figure 3).

Important microbiome pathways and ECs to 1mo WAZ are also involved in vitamin B, starch metabolism, and signalling. For example, flavin biosynthesis pathway III (vitamin B2 synthesis) predicted an increase of 0.5 sd (Supplementary Figure 9). Cellulase abundance predicted ~0.5 sd increase above the 87^th^ abundance percentile. D-fructuronate degradation and sucrose degradation pathway III predicted 1 sd and 0.2 sd increase in WAZ, respectively (Supplementary Figure 9). While enzymes involved in signalling predicted 0.2 sd increase (e.g cytidine deaminase) or >0.2 sd decrease (e.g. GMP reductase) (Supplementary Figure 8).

### Important XGBoost variables for LAZ at 1mo

Gestational age, maternal height and MUAC were also among the most important predictors of LAZ at 1mo; however, *Anaerotruncus sp* CAG 528 exhibited a higher relative importance score than maternal height and MUAC (Supplementary Figure 10). Resistant-starch degraders also figured prominently in the set of taxa important to LAZ, as well as the dietary fiber fermenting *Prevotella copri* [73,74] (Supplementary Figure 10). Some important microbiome functions to 1mo LAZ were also important to WAZ and are also involved in starch metabolism, signalling and vitamin B metabolism (Supplemental Figures 11 and 121). For example, holo- [acyl-carrier-protein] synthase (vitamin B5 and Coenzyme A metabolism) and 4-phosphoerythronate dehydrogenase (vitamin B6 metabolism) predicted a 1 sd and 0.2 sd increase in LAZ, respectively, below the 25^th^ abundance percentile (Supplemental Figure 10).

### Important XGBoost variables for gestational age

The most important predictors of gestational age were all microbiome taxa or microbiome diversity metrics, except for trimester of specimen collection and maternal MUAC (Supplemental Figure 13). As with other outcomes, several important taxa are associated with resistant-starch degradation, including members of the *Ruminococcaceae, Lachnospiraceae*, and *Eubacteriaceae* families [69,70] and dietary fiber, including *Prevotella copri* [73,74] which predicted a 0.5 week decrease in gestation (Supplemental Figure 13). Another important taxonomic predictor of gestational age was *Slackia isoflavoniconvertens* (1 week increase in gestational age through the range of observed abundances). Species richness also predicted ~0.5 week shorter gestation in enzyme and pathway models (Supplementary Figure 14 and 15).

## Discussion

In this study of 207 mothers and their infants in rural Zimbabwe where there is a high prevalence of preterm birth and LBW, we investigated the relationship between pregnancy microbiome characteristics and gestational age, birth weight or neonatal growth. Taxonomic features of the maternal microbiome alone predicted birth weight (pseudo-R^2^=0·23), 1mo WAZ (pseudo-R^2^=0·21) and LAZ (pseudo-R^2^=0·11) as accurately as models that combined microbiome features with sociodemographic and epidemiologic variables, pointing to the importance of the pregnancy microbiome. Gestational age was predicted less accurately (pseudo-R^2^=0·05). Prediction accuracy for all outcomes was improved when functional enzyme relative abundances were used (pseudo-R^2^: 0·25-0·37), demonstrating the potential value of whole metagenome shotgun sequencing for investigating the human microbiome and health. There were only modest global differences between mothers allocated to the WASH versus the non-WASH arms of the SHINE trial, as measured by β-diversity, and only few differences in taxon abundances after FDR p-value adjustment. In prior analyses, the WASH intervention also had only a modest impact on pathogen carriage and diarrhea in SHINE infants [75]. Surprisingly, the same was true for maternal HIV-status and pregnancy trimester in this substudy.

The maternal fecal microbiome of rural Zimbabwean mothers was highly enriched for metabolizers of resistant-starch, including *Ruminococcus bromii* and *Faecalibacterium prausnitizii*, most likely driven by the daily consumption of maize by all mothers. These starch-degraders and producers of SCFAs were among the most abundant and most prevalent. Starch-degraders release energy from dietary polysaccharides that are not processed by host enzymes, providing an important nutrient-harvesting function for the host. Although common to many human intestinal microbiomes, they showed important associations with birth weight and neonatal growth in this study population. *Prevotella copri* and *Eubacterium rectale* were also highly abundant species. *P. copri* tends to be more abundant in the microbiomes of populations living non-Westernized lifestyles [76], and have been associated with high fibre diets. *E. rectale* has also been shown to increase, along with Eubacterium-derived butyrate, in subjects given resistant-starch from maize [77]. As with *R. bromii*, and other resistant starch-degraders, *P. copri* and *E. rectale* are likely more abundant due to dietary selection.

However, the direction of taxon abundance relationships with birth weight and neonatal growth was quite varied. Notably, greater relative abundance of *Butyrivibrio sp* CAG 318 and *Roseburia intestinalis,* predicted larger birth weight, while *Catenibacterium mitsuokai* predicted greater 1mo WAZ. These bacteria are butyrate producers with the capacity to degrade the plant fibers hemicellulose [78,79], mannan [80], and inulin [71,81], respectively. Starch-metabolizing, SCFA-producing microbes can regulate gestational weight gain [82], and may impact fetal growth [83]. However, increasing abundance of several other starch-degrading taxa predicted worse outcomes. Different species of bacteria do appear to prefer different sources of dietary fiber [84], suggesting that the potential benefits of SCFA-producing primary starch-degraders may partly depend on both the dietary-fiber source consumed and the resident gut species. The improvement in outcomes predicted by an increase in some taxa may reflect a response of these particular microbes to specific dietary fibers consumed by some mothers in this population. The abundance of specific taxa may also be a marker of dietary deprivation that could explain decreases in birth weight or neonatal growth. Microbiome evenness was also associated with increasing birth weight and 1mo WAZ. Evenness measures the uniformity of the abundance of taxa present in a microbiome. A less even microbiome may reflect selection for only a few predominant taxa by low dietary diversity, resulting in lower birth weight and neonatal WAZ.

Also, there were remarkably few Bacteroidetes, aside from *Prevotella spp*, that emerged in these analyses. The lack of *Bacteroides spp* specifically could indicate a dietary deficiency, as these are associated with a diet rich in meat or fat [73] and may reflect dietary or functional microbiome differences that contribute to pregnancy outcomes and neonatal growth.

Several pregnancy gut microbiome metabolic pathways and enzymes were also important predictors of birth weight, WAZ and LAZ. These broadly reflected starch metabolism, vitamin B metabolism, signalling and environmental sensing. For example, greater relative abundance of the microbiome enzymes for plant fiber degradation (e.g. cellulase, neopullulanase) and pathways for sucrose and fiber degradation (e.g. D-fructuronate degradation) predicted greater birth weight and neonatal growth. These provide further evidence that a microbiome with an increased capacity to degrade the maize-rich, plant-based Zimbabwean diet, is beneficial for healthier growth. Also, greater abundance of histidine kinase predicted larger birth weight. Histidine kinases are important in bacterial sensing, signal transduction and energy utilization [85]. Bacteria that possess many histidine kinases are generally able to adapt to a variety of environmental stimuli [86]. Histidine kinase abundance may reflect a microbiome that is more robust to unstable nutrient availability. In contrast, glycogen synthase and bacterial functions involved in purine metabolism (e.g. ppGpp synthesis, GMP reductase) predicted decreasing birth weight; while signalling enzymes involved in biofilm dispersal (e.g. cytidine deaminase) predicted increasing WAZ. Glycogen synthesis is an important strategy for bacterial survival and persistence in conditions of fluctuating nutrient availability [87], and facilitates transition into a biofilm state in response to bacterial nutrient starvation [88]. Purine and pyrimidine salvage pathways are also potential markers of biofilm formation and dispersal to a planktonic state [89], [90], [91], [92], [93]. The role of these microbial mechanisms in pregnancy outcomes requires further investigation, but these data suggest that a monotonous, nutrient-poor diet, may induce a starvation response in some maternal gut microbiomes that display characteristics of biofilm formation. Microbiome functions related to vitamin B biosynthesis pathways predicted greater birth weight, WAZ or LAZ. This may reflect varied microbiome capacities for vitamin B metabolism in response to deficiencies in the host diet.

An important taxonomic predictor of longer gestation was *Slackia isoflavoniconvertens*. When present, *S. isoflavoniconvertens* converts dietary isoflavones, largely found in soy [94], to equol [95,96]. Equol has been found to reduce anxiety and depressive behaviour in rodent models [97], [98], [99], and humans [99]. This mechanism could influence gestation length [100,101]. In contrast, *Prevotella copri* predicted reduced gestation. *P. copri* has been associated with increased gut inflammation, damage [102] and bacterial translocation [103]. Host inflammation drives increased blood pressure [104], [105], [106], which is an important risk factor for preterm birth. The inflammatory contribution of the maternal gut microbiome may contribute to reduced gestation in this sub-Saharan African population. These results should be interpreted with caution as the gestational age outcome model exhibited the weakest performance metrics.

Overall, pathogen carriage was low, aside from *Brachyspira* which is associated with intestinal spirochetosis [92]. However, four spirochetes [107] were carried by many mothers and *Treponema berlinense* predicted lower 1mo WAZ. Treponemes have been found in the microbiota of non-Western populations [108], [109], [110] and may represent a critical member of an evolutionarily intact intestinal microbiome, similar to *Helicobacter pylori*, another spirochete, which typically resides in the stomach. The contribution of non-pathogenic *Treponema sp* to intestinal microbiome function is unknown. In a macaque model that tested the impact of a high- versus low-fat diet on maternal and offspring microbiota, the largest change was a significant reduction in non-pathogenic *Treponema spp* (*T. berlinense, T. porcinum* and *T. parvum*), in dams administered a high-fat diet, regardless of obese or lean status. The authors suggested that these *Treponema spp* benefited from a low fat, plant-rich diet (corn and soy bean) [111].

There are notable limitations to our analyses. A large fraction of the sequenced reads was un-assignable. The fact that there was a large proportion of unknown or unidentifiable DNA in these samples is not surprising, as microbiome data from LMICs are under-represented in reference databases. However, the sheer scale of un-assignable DNA reads was an important observation and is rarely reported in microbiome studies. *E. coli* was common and predicted decreasing birth weight, but specific *E. coli* pathotypes could not be distinguished by MetaPhlAn3, limiting our ability to assess the impact of these pathogens. Differences in microbiome characteristics could also be influenced by dietary deprivation. However, wealth quintile was not an important predictor in any models.

In conclusion, our analyses illustrate that the pregnancy fecal microbiome, primarily the abundance of resistant-starch degraders, is an important contributor to birth weight and neonatal growth, and to a lesser extent gestational age, in infants of rural Zimbabwean mothers who consume a diet high in maize. The functional capacity for starch metabolism and environmental sensing may be important microbiome mechanisms during pregnancy; while a nutrient-poor diet dominated by maize may promote microbiome biofilm formation in response to nutrient starvation in the gut, with potentially detrimental consequences for the infant. Future work is warranted to confirm these mechanisms and explore whether interventions to facilitate starch degradation and improve dietary diversity can improve birth weight and neonatal growth in populations with a monotonous starch-rich diet.

## Contributors

ARM, LES, RJS, LHM, JHH and AJP conceptualized and designed the study. KM, RN, BC, FDM, NVT, JT, and BM collected data and biospecimens. HMG, IB, SKG, RCR, FF and LC processed biospecimens. TJE performed bioinformatics. ARM, TJE and EKG analyzed and interpreted data. ARM and TJE drafted the manuscript. EKG substantially revised the manuscript. ARM, AJP and JHH verified the underlying data. All authors read and approved the final version of the manuscript.

## Declaration of Interests

RCR declares monetary support from Nestle Nutrition Institute for conference attendance (April 2019), outside the submitted work. TJE was paid a scientific consulting fee in relation to the analysis of the data presented here by Zvitambo Instititute for Maternal and Child Health Research. All other authors have no interests to declare.

## Declaration of Competing Interest

None.

## Appendix Supplementary materials

Image, application 1

Image, application 1

Image, application 1
