# Supplementary material for: Maternal fecal microbiome predicts gestational age, birth weight and neonatal growth in rural Zimbabwe
Source: eBioMedicine. 2021 Jun 15;68:103421. doi: 10.1016/j.ebiom.2021.103421 (PMC8217692; doi:10.1016/j.ebiom.2021.103421)
Supplement: Supplementary file 1 [file mmc1.docx]

**Supplementary Table 1. Grid search hyperparameters for all XGBoost models**

| **XGBoost Parameter** | **Range*** |
| --- | --- |
| init_points | {10} |
| n_initer | {50} |
| max_depth | { 2,3,4,5 } |
| log10_ntrees | [ 1.5, 2.5 ] |
| log10_learn_rate | [ log10( 0.1 ), log10( 0.3 ) ] |
| sample_rate | [ 0.8, 1 ] |
| col_sample_rate | [ 0.3, 1 ] |
| col_sample_rate_per_tree | [ 0.5, 1.0 ] |
| min_child_weight | { 1,2,3 } |

*{} indicate a set and [] indicate a range of real values.

**Supplementary Table 2. Diet consumption on a normal day among the sub-sample of mothers included in the microbiome sub-study**

| **Food Item** | **SHINE mothers included in the microbiome sub-study**  **n = 207** | | |
| --- | --- | --- | --- |
|  | **HIV-positive**  **n = 97** |  | **HIV-negative**  **n = 110** |
| Cereals (e.g. sadza, maize, rice, wheat, other local grains) | 97 (100%) |  | 110 (100%) |
| Vitamin A-rich vegetables and tubers (e.g. pumpkin, carrot, sweet potatoes) | 12 (13%) |  | 15 (14%) |
| White tubers and roots (e.g. potatoes, white yams) | 8 (9%) |  | 17 (16%) |
| Dark green leafy vegetables (e.g. spinach, rape, covo) | 62 (67%) |  | 74 (67%) |
| Other vegetables (e.g. squash, cabbage, onion) | 55 (60%) |  | 61 (56%) |
| Vitamin A-rich fruits (e.g. ripe mangoes, pawpaw) | 11 (12%) |  | 15 (14%) |
| Other fruits (e.g. bananas, apples, guavas) | 28 (30%) |  | 27 (25%) |
| Any beef, goat, lamb, chicken, duck, organ meats | 42 (46%) |  | 56 (51%) |
| Eggs (e.g. from any bird) | 14 (15%) |  | 17 (16%) |
| Fish (e.g. fresh, dried or shellfish) | 23 (25%) |  | 15 (14%) |
| Any foods made from beans, peas or lentils | 22 (24%) |  | 24 (22%) |
| Nuts (e.g. peanuts) | 32 (35%) |  | 31 (28%) |
| Cheese, yoghurt, milk or other milk products | 30 (33%) |  | 45 (42%) |
| Foods made with oil, fat or butter | 83 (90%) |  | 96 (87%) |
| Sweets (e.g. sugar, molasses, honey) | 67 (73%) |  | 82 (75%) |
| Spices, condiments | 80 (88%) |  | 99 (90%) |
| Tea/coffee other beverages | 67 (74%) |  | 77 (71%) |

**Supplementary Table 3. Influence of baseline variables on maternal fecal microbiota during and after pregnancy**

| **Variable category** | **Specimen Visit** | **Taxon** | **Independent variable** | **Coefficient** | **Standard Error** | **Test Statistic** | **P-value** | **Relative abundance ratio** | **Q-value** |
| --- | --- | --- | --- | --- | --- | --- | --- | --- | --- |
| **HIV-related** | 1m post-partum | *Treponema_berlinense* | HIV status – negative | 1.541 | 0.351 | 4.391 | 0.000 | 4.669 | 0.003 |
|  |  |  |  |  |  |  |  |  |  |
| **Birth and delivery-related** | Pregnancy | *Enterococcus_faecium* | Non-institutional delivery | 1.775 | 0.429 | 4.138 | 0.000 | 5.902 | 0.007 |
|  | Pregnancy | *Anaerostipes_hadrus* | Forceps or vacuum delivery | 1.943 | 0.389 | 4.992 | 0.000 | 6.976 | 0.000 |
|  | Pregnancy | *Methanobrevibacter_smithii* | Forceps or vacuum delivery | 2.356 | 0.597 | 3.947 | 0.000 | 10.545 | 0.009 |
|  | Pregnancy | *Haemophilus_parainfluenzae* | Caesarean section | 2.174 | 0.273 | 7.962 | 0.000 | 8.790 | 0.000 |
|  | Pregnancy | *Treponema_berlinense* | Caesarean section | 1.474 | 0.423 | 3.480 | 0.001 | 4.365 | 0.042 |
|  | Pregnancy | *Alistipes_shahii* | Normal birth weight infant | -1.779 | 0.276 | -6.435 | 0.000 | 0.169 | 0.000 |
|  | Pregnancy | *Bacteroides_fragilis* | Normal birth weight infant | -1.414 | 0.391 | -3.613 | 0.000 | 0.243 | 0.029 |
|  |  |  |  |  |  |  |  |  |  |
| **Wealth + Education** | 1m post-partum | *Brachyspira_pilosicoli* | Wealth index score | -0.268 | 0.064 | -4.184 | 0.000 | 0.765 | 0.006 |
|  | Pregnancy | *Brachyspira_pilosicoli* | Primary school vs. higher | 3.031 | 0.604 | 5.017 | 0.000 | 20.720 | 0.000 |
|  | Pregnancy | *Roseburia_sp_CAG_471* | Primary school vs. higher | -0.847 | 0.220 | -3.846 | 0.000 | 0.429 | 0.011 |
|  |  |  |  |  |  |  |  |  |  |
| **Maternal characteristics** | 1m post-partum | *Klebsiella_variicola* | Maternal age | 0.070 | 0.019 | 3.644 | 0.000 | 1.072 | 0.047 |
|  | Pregnancy | *Brachyspira_pilosicoli* | Maternal height | 0.107 | 0.022 | 4.814 | 0.000 | 1.113 | 0.005 |
|  | Pregnancy | *Desulfovibrio_piger* | Maternal MUAC | 0.213 | 0.054 | 3.928 | 0.000 | 1.238 | 0.014 |
|  | Pregnancy | *Klebsiella_pneumoniae* | Maternal MUAC | 0.195 | 0.021 | 9.130 | 0.000 | 1.216 | 0.000 |
|  | Pregnancy | *Klebsiella_quasipneumoniae* | Maternal MUAC | 0.182 | 0.022 | 8.218 | 0.000 | 1.199 | 0.000 |
|  | Pregnancy | *Klebsiella_variicola* | Maternal MUAC | 0.160 | 0.024 | 6.787 | 0.000 | 1.173 | 0.000 |
|  | 1m post-partum | *Klebsiella_variicola* | Parity | 0.500 | 0.121 | 4.119 | 0.000 | 1.648 | 0.008 |
|  | Pregnancy | *Clostridium_sp_CAG_590* | Coping skills index | 0.031 | 0.008 | 3.639 | 0.000 | 1.031 | 0.025 |
|  | Pregnancy | *Eubacterium_sp_CAG_786* | Coping skills index | 0.061 | 0.009 | 6.559 | 0.000 | 1.062 | 0.000 |
|  | 1m post-partum | *Firmicutes_bacterium_CAG_110* | Coping skills index | 0.051 | 0.009 | 5.654 | 0.000 | 1.052 | 0.000 |
|  | 1m post-partum | *Roseburia_sp_CAG_303* | Coping skills index | 0.093 | 0.016 | 5.665 | 0.000 | 1.098 | 0.000 |
|  | 1m post-partum | *Ruminococcus_bicirculans* | Coping skills index | 0.024 | 0.007 | 3.716 | 0.000 | 1.024 | 0.018 |
|  | Pregnancy | *Streptococcus_salivarius* | Possible Depression | 1.700 | 0.253 | 6.715 | 0.000 | 5.472 | 0.000 |
|  |  |  |  |  |  |  |  |  |  |
| **Percent human DNA in specimen** | Pregnancy | *Agathobaculum_butyriciproducens* | Percent human DNA | 0.085 | 0.017 | 5.086 | 0.000 | 1.088 | 0.000 |
|  | Pregnancy | *Alistipes_shahii* | Percent human DNA | 1.545 | 0.402 | 3.845 | 0.000 | 4.689 | 0.001 |
|  | Pregnancy | *Bacteroides_caccae* | Percent human DNA | 1.507 | 0.530 | 2.842 | 0.005 | 4.514 | 0.028 |
|  | Pregnancy | *Bacteroides_vulgatus* | Percent human DNA | 0.187 | 0.023 | 7.955 | 0.000 | 1.205 | 0.000 |
|  | Pregnancy | *Brachyspira_pilosicoli* | Percent human DNA | 1.228 | 0.150 | 8.178 | 0.000 | 3.415 | 0.000 |
|  | Pregnancy | *Clostridium_disporicum* | Percent human DNA | 1.400 | 0.148 | 9.452 | 0.000 | 4.056 | 0.000 |
|  | Pregnancy | *Clostridium_sp_CAG_510* | Percent human DNA | 0.895 | 0.201 | 4.456 | 0.000 | 2.447 | 0.000 |
|  | Pregnancy | *Eubacterium_eligens* | Percent human DNA | 0.265 | 0.088 | 3.023 | 0.003 | 1.303 | 0.018 |
|  | Pregnancy | *Eubacterium_sp_CAG_38* | Percent human DNA | 0.137 | 0.012 | 11.147 | 0.000 | 1.147 | 0.000 |
|  | Pregnancy | *Haemophilus_parainfluenzae* | Percent human DNA | 0.107 | 0.017 | 6.248 | 0.000 | 1.113 | 0.000 |
|  | Pregnancy | *Intestinibacter_bartlettii* | Percent human DNA | 0.064 | 0.024 | 2.738 | 0.007 | 1.066 | 0.038 |
|  | Pregnancy | *Lachnospira_pectinoschiza* | Percent human DNA | 0.105 | 0.017 | 6.078 | 0.000 | 1.111 | 0.000 |
|  | Pregnancy | *Parabacteroides_goldsteinii* | Percent human DNA | 2.550 | 0.555 | 4.595 | 0.000 | 12.802 | 0.000 |
|  | Pregnancy | *Prevotella_sp_885* | Percent human DNA | 0.338 | 0.076 | 4.419 | 0.000 | 1.402 | 0.000 |
|  | Pregnancy | *Prevotella_sp_CAG_1092* | Percent human DNA | 0.360 | 0.072 | 4.999 | 0.000 | 1.433 | 0.000 |
|  | Pregnancy | *Prevotella_sp_CAG_520* | Percent human DNA | 0.349 | 0.084 | 4.144 | 0.000 | 1.418 | 0.000 |
|  | Pregnancy | *Prevotella_stercorea* | Percent human DNA | 0.374 | 0.090 | 4.175 | 0.000 | 1.454 | 0.000 |
|  | Pregnancy | *Roseburia_sp_CAG_182* | Percent human DNA | 0.726 | 0.215 | 3.376 | 0.001 | 2.066 | 0.006 |
|  | Pregnancy | *Ruminococcus_torques* | Percent human DNA | 0.080 | 0.020 | 3.903 | 0.000 | 1.083 | 0.001 |
|  | Pregnancy | *Veillonella_atypica* | Percent human DNA | 0.643 | 0.220 | 2.925 | 0.004 | 1.902 | 0.023 |
|  | Pregnancy | *Veillonella_sp_T11011_6* | Percent human DNA | 0.823 | 0.185 | 4.448 | 0.000 | 2.277 | 0.000 |
|  | 1m post-partum | *Alistipes_shahii* | Percent human DNA | 6.797 | 0.860 | 7.901 | 0.000 | 895.437 | 0.000 |
|  | 1m post-partum | *Blastocystis_sp_subtype_1* | Percent human DNA | 7.714 | 1.157 | 6.670 | 0.000 | 2240.374 | 0.000 |
|  | 1m post-partum | *Oscillibacter_sp_57_20* | Percent human DNA | 0.085 | 0.025 | 3.426 | 0.001 | 1.089 | 0.032 |
|  | 1m post-partum | *Turicibacter_sanguinis* | Percent human DNA | 1.598 | 0.263 | 6.067 | 0.000 | 4.944 | 0.000 |
|  | 1m post-partum | *Veillonella_parvula* | Percent human DNA | 0.400 | 0.092 | 4.338 | 0.000 | 1.492 | 0.001 |
|  |  |  |  |  |  |  |  |  |  |
| **Baseline household WASH + WASH arm** | 1m post-partum | *Roseburia_sp_CAG_303* | Any latrine at baseline | 1.164 | 0.237 | 4.905 | 0.000 | 3.204 | 0.000 |
|  | 1m post-partum | *Brachyspira_sp_CAG_700* | Time to water | 0.017 | 0.003 | 5.281 | 0.000 | 1.017 | 0.000 |
|  | 1m post-partum | *Firmicutes_bacterium_CAG_791* | Time to water | 0.017 | 0.004 | 4.819 | 0.000 | 1.017 | 0.000 |
|  | 1m post-partum | *Veillonella_atypica* | Time to water | 0.045 | 0.007 | 6.551 | 0.000 | 1.046 | 0.000 |
|  | 1m post-partum | *Bifidobacterium_longum* | Treated water – yes | 1.629 | 0.236 | 6.910 | 0.000 | 5.100 | 0.000 |
|  | 1m post-partum | *Haemophilus_sp_HMSC71H05* | Treated water – yes | 1.486 | 0.193 | 7.681 | 0.000 | 4.420 | 0.000 |
|  | 1m post-partum | *Akkermansia_muciniphila* | WASH arm assignment | -0.992 | 0.286 | -3.468 | 0.001 | 0.371 | 0.042 |
|  | 1m post-partum | *Brachyspira_sp_CAG_700* | WASH arm assignment | 1.938 | 0.304 | 6.368 | 0.000 | 6.945 | 0.000 |

**Supplementary Table 4. Epidemiologic variables associated with infant birthweight, LAZ and WAZ at 1mo of age in multivariable linear regression models**

| **Factor** |  | **Model parameters** | | | |
| --- | --- | --- | --- | --- | --- |
|  |  | **Coefficient (SE)** |  | **p-value** |  |
| Birth weight (kg) |  |  |  |  |  |
| Female |  | 0.12 (1.02) |  | 0.350 |  |
| Gestational age (days) |  | 0.003 (0.001) |  | 0.010 |  |
| Maternal mid-upper arm circumference (cm) |  | 0.02 (0.01) |  | 0.040 |  |
| Maternal height (cm) |  | 0.01 (0.006) |  | 0.050 |  |
|  |  |  |  |  |  |
| LAZ (z-score) |  |  |  |  |  |
| Female |  | 0.21 (0.18) |  | 0.250 |  |
| Gestational age (days) |  | 0.01 (0.004) |  | 0.002 |  |
| Maternal mid-upper arm circumference (cm) |  | 0.05 (0.03) |  | 0.090 |  |
| Maternal height (cm) |  | 0.03 (0.016) |  | 0.060 |  |
| WASH arm |  | 0.52 (0.18) |  | 0.005 |  |
|  |  |  |  |  |  |
| WAZ (z-score) |  |  |  |  |  |
| Female |  | 0.03 (0.21) |  | 0.870 |  |
| Gestational age (days) |  | 0.003 (0.02) |  | 0.490 |  |
| Maternal mid-upper arm circumference (cm) |  | 0.09 (0.03) |  | 0.009 |  |
| Maternal height (cm) |  | 0.02(0.02) |  | 0.220 |  |

SE, standard error; Kg, kilograms; cm, centimeters, WASH, water, sanitation, and hygiene intervention arm.

**Supplementary Table 5. XGBoost performance parameters for maternal microbiome relative abundance and gestational age, fetal or neonatal growth.**

| **Microbiome variables** | **Outcome variable** | **Variable set*** | **pseudo-R^2^**** | **Root Mean square error (RMSE)** | **Mean absolute error (MAE)** |
| --- | --- | --- | --- | --- | --- |
| Species | Gestational Age | 1 | -0.08 | 3.61 | 2.39 |
| Species | Gestational Age | 2 | 0.05 | 3.38 | 2.23 |
| Species | Gestational Age | 3 | 0.11 | 3.28 | 2.24 |
| EC | Gestational Age | 1 | 0.28 | 2.95 | 1.92 |
| EC | Gestational Age | 2 | 0.25 | 3.01 | 2.01 |
| EC | Gestational Age | 3 | 0.47 | 2.52 | 1.76 |
| Pathway | Gestational Age | 1 | 0.07 | 3.25 | 2.15 |
| Pathway | Gestational Age | 2 | 0.17 | 3.16 | 2.11 |
| Pathway | Gestational Age | 3 | 0.06 | 3.36 | 2.25 |
| Species | Birth weight | 1 | 0.23 | 0.44 | 0.34 |
| Species | Birth weight | 2 | 0.20 | 0.45 | 0.35 |
| Species | Birth weight | 3 | 0.06 | 0.48 | 0.38 |
| EC | Birth weight | 1 | 0.37 | 0.40 | 0.30 |
| EC | Birth weight | 2 | 0.42 | 0.38 | 0.28 |
| EC | Birth weight | 3 | 0.35 | 0.40 | 0.31 |
| Pathway | Birth weight | 1 | 0.25 | 0.43 | 0.33 |
| Pathway | Birth weight | 2 | 0.29 | 0.42 | 0.34 |
| Pathway | Birth weight | 3 | 0.29 | 0.42 | 0.33 |
| Species | WAZ 1mo | 1 | 0.21 | 1.33 | 0.98 |
| Species | WAZ 1mo | 2 | 0.28 | 1.27 | 0.98 |
| Species | WAZ 1mo | 3 | 0.10 | 1.41 | 1.04 |
| EC | WAZ 1mo | 1 | 0.25 | 1.29 | 0.92 |
| EC | WAZ 1mo | 2 | 0.14 | 1.38 | 0.99 |
| EC | WAZ 1mo | 3 | 0.36 | 1.19 | 0.90 |
| Pathway | WAZ 1mo | 1 | 0.17 | 1.35 | 0.98 |
| Pathway | WAZ 1mo | 2 | 0.19 | 1.34 | 0.98 |
| Pathway | WAZ 1mo | 3 | 0.14 | 1.38 | 1.00 |
| Species | LAZ 1mo | 1 | 0.11 | 1.26 | 0.94 |
| Species | LAZ 1mo | 2 | 0.08 | 1.28 | 0.95 |
| Species | LAZ 1mo | 3 | 0.07 | 1.29 | 0.94 |
| EC | LAZ 1mo | 1 | 0.27 | 1.14 | 0.84 |
| EC | LAZ 1mo | 2 | 0.24 | 1.16 | 0.86 |
| EC | LAZ 1mo | 3 | 0.17 | 1.21 | 0.90 |
| Pathway | LAZ 1mo | 1 | 0.10 | 1.27 | 0.96 |
| Pathway | LAZ 1mo | 2 | 0.01 | 1.33 | 1.00 |
| Pathway | LAZ 1mo | 3 | 0.07 | 1.29 | 0.96 |

*1, microbiome relative abundance + diversity metrics + epidemiologic variables; 2, microbiome relative abundance + diversity metrics; 3, microbiome relative abundance.

**R-squared between observed and model predicted outcome values.

**
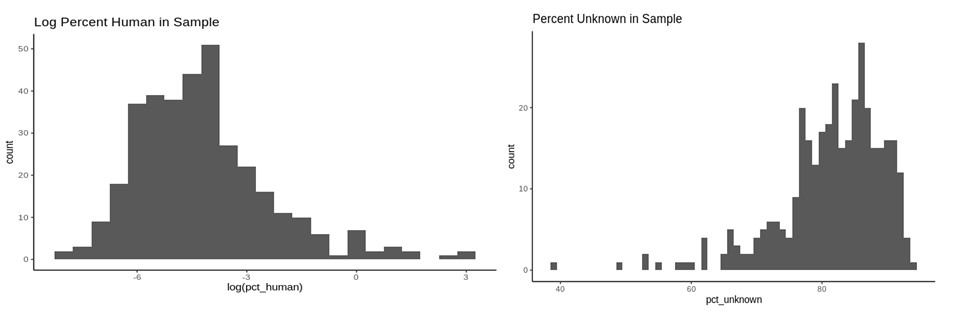
**

**Supplementary Figure 1. Distributions of (log-transformed) percent human DNA and percent non-annotatable sequencing reads from maternal microbiome datasets.**

**
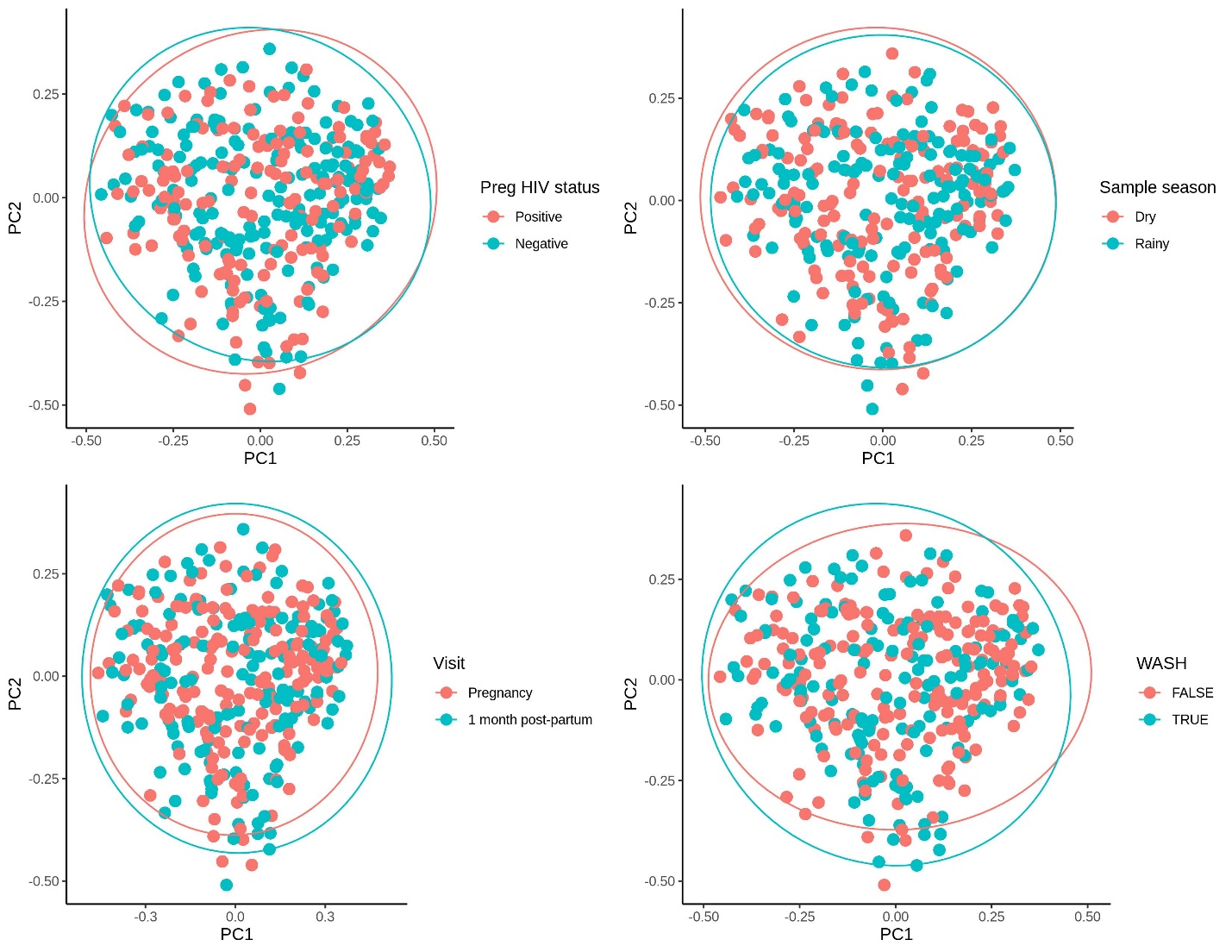
**

**Supplementary Figure 2. Principal coordinate analysis based on Bray-Curtis dissimilarity index for maternal fecal microbiomes by maternal HIV status, specimen collection visit, season of specimen collection, or randomized WASH arm.** 95% confidence ellipses are provided.

**
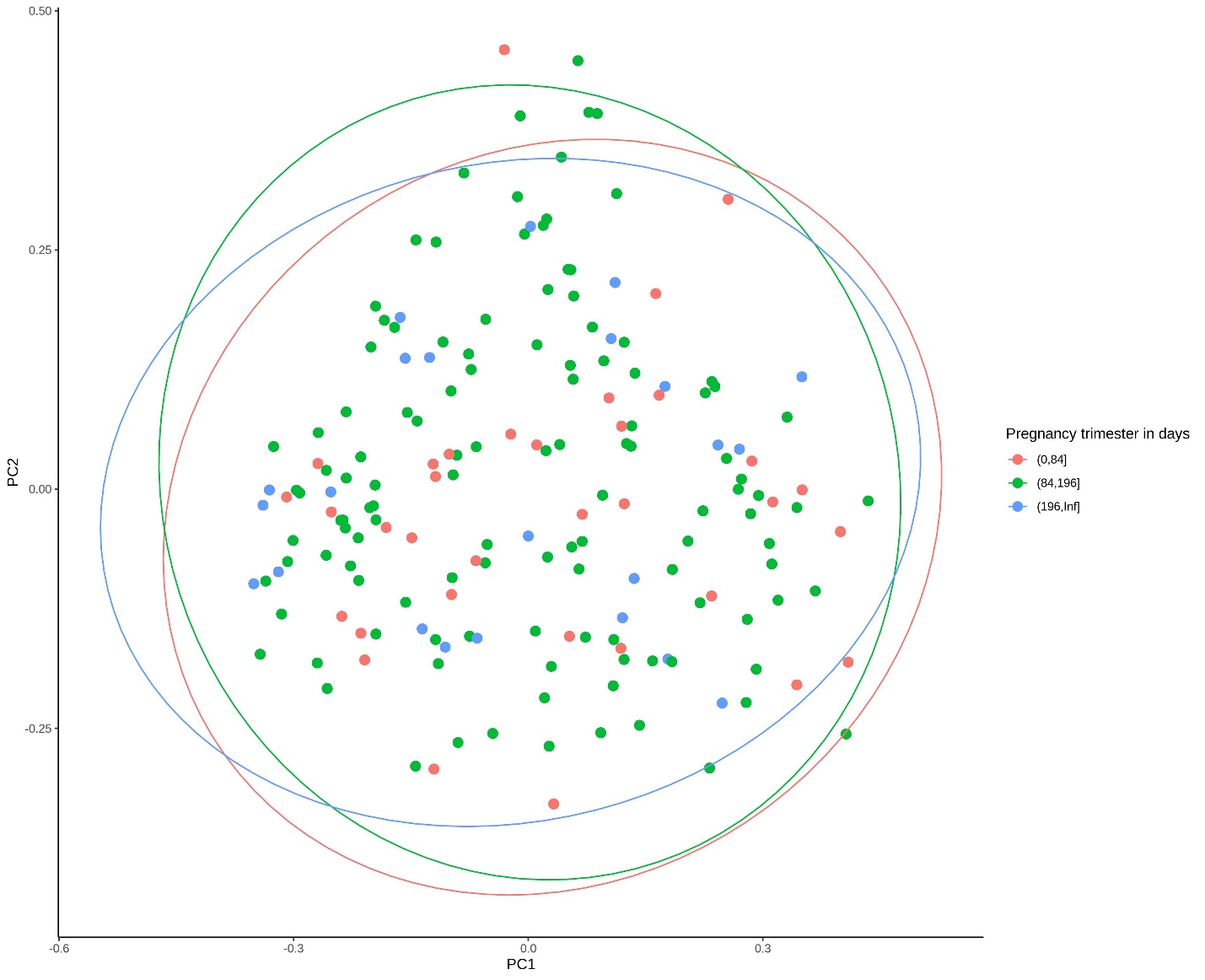
**

**Supplementary Figure 3. Principal coordinate analysis based on Bray-Curtis dissimilarity index for maternal fecal microbiomes by trimester of Pregnancy.** 95% confidence ellipses are provided.


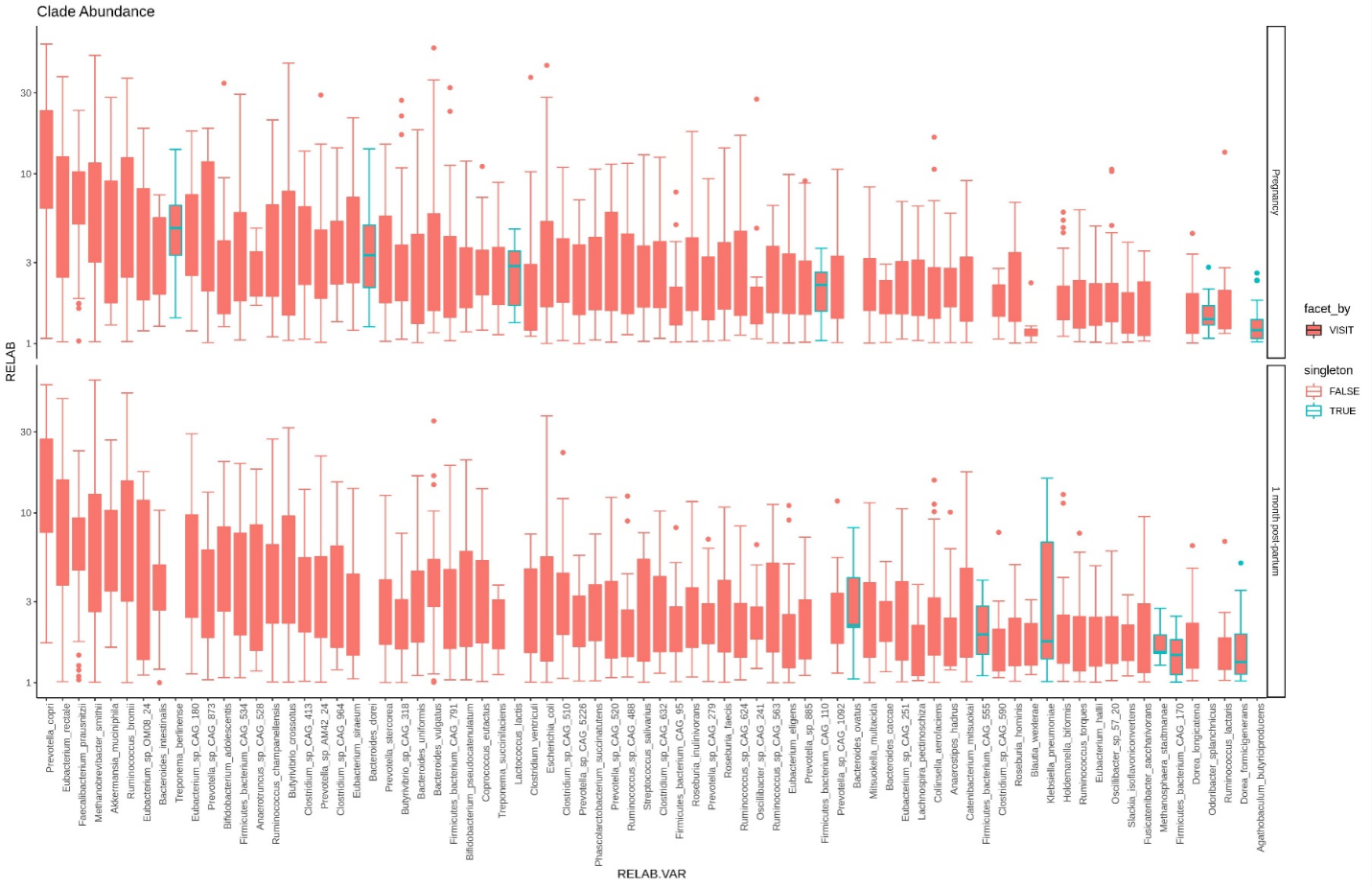


**Supplementary Figure 4. The distribution of prevalent (present in ≥ 5% of SHINE mothers) and abundant (present at ≥ 1% relative abundance) taxa in the gestational visit (top) and in the one-month post-partum (bottom) visit, as defined by MetaPhlAn3.** Blue outlined boxes are taxa that are present at only one visit.


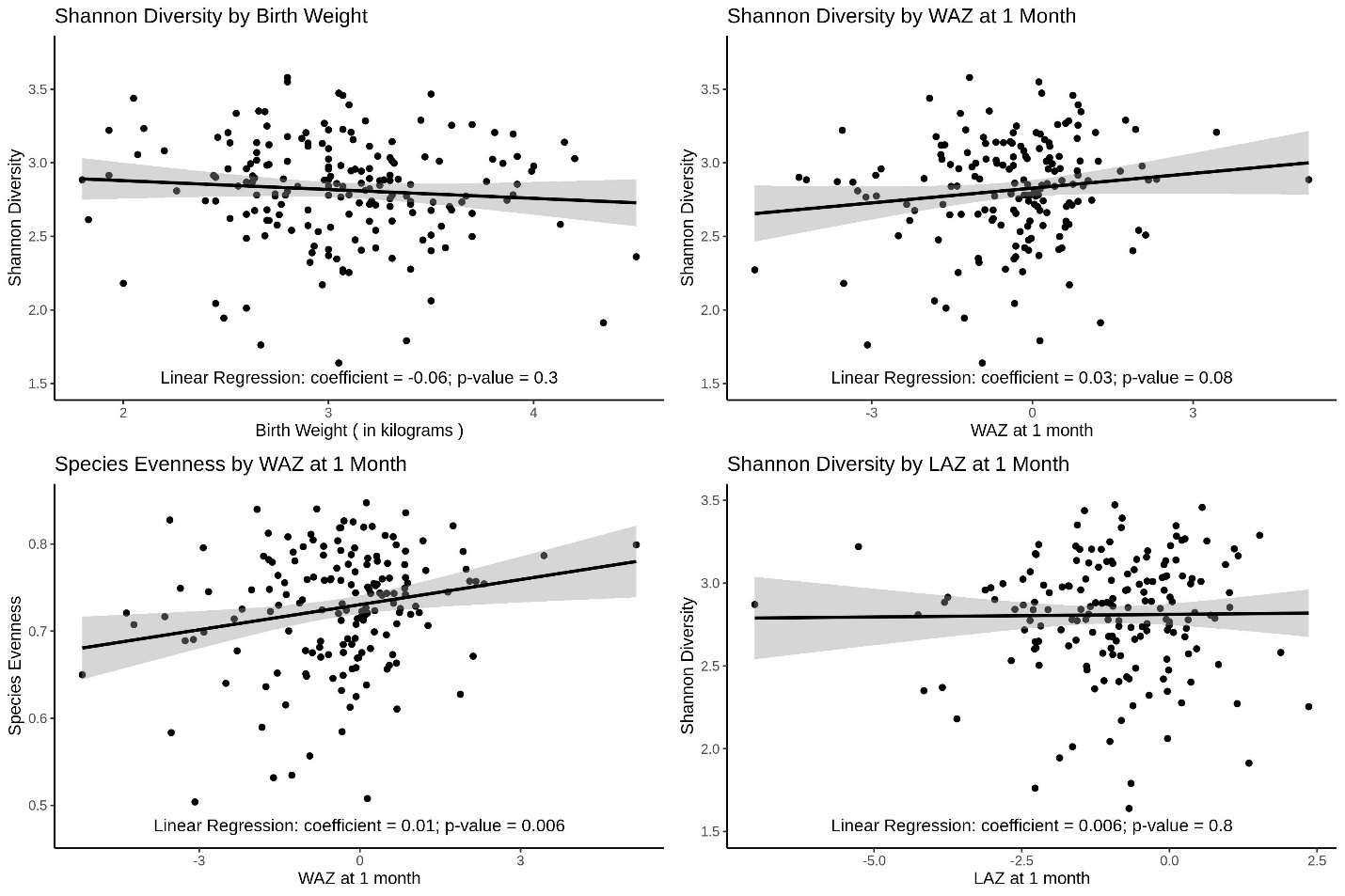


**Supplementary Figure 5. Relationship between α-diversity measures and select infant growth outcomes. Relationship between α-diversity measures and select infant growth outcomes.** A single dataset with very low (> 4 s.d.) α-diversity measures was removed from the analyses.


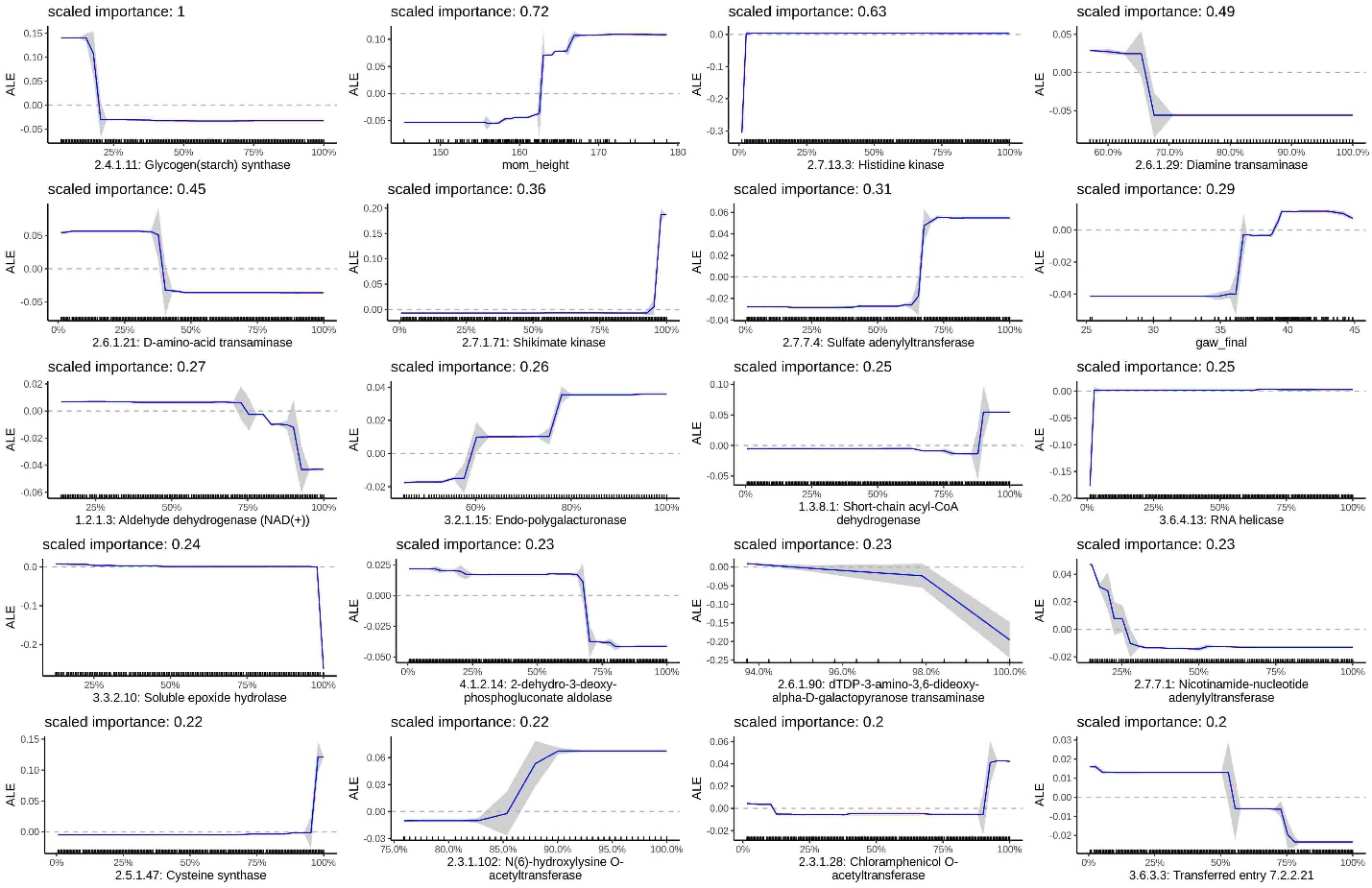


**Supplementary Figure 6. Relationships between infant birth weight in kg, maternal characteristics, and maternal gut microbiome Enzyme Commission Categories (EC) relative abundance.** The top 20 predictors of infant birth weight by variable importance score are shown. For microbiome abundances, the x-axis represents the percentile of the abundance distribution. Epidemiologic and microbiome diversity variables are on the original scale. Tick marks on the x-axis are a rug plot of individual feature abundance percentiles. ALEs were generated using the *ALEplot* package and were plotted using *ggplot2*. Standard deviations (sd) were calculated per increment in microbiome feature and were used to calculate and plot increment-wise 95% confidence intervals as the average change in the outcome ±1.96(sd/sqrt(n)), where n is the number of observed feature values, and sd is the standard deviation of the change in the outcome variable in an interval. gaw_final, gestational age; mom_height, maternal height in centimeters; mom_muac, maternal mid-upper arm circumference in millimeters; pct_human, percent human reads; pct_unknown, percent unknown reads.


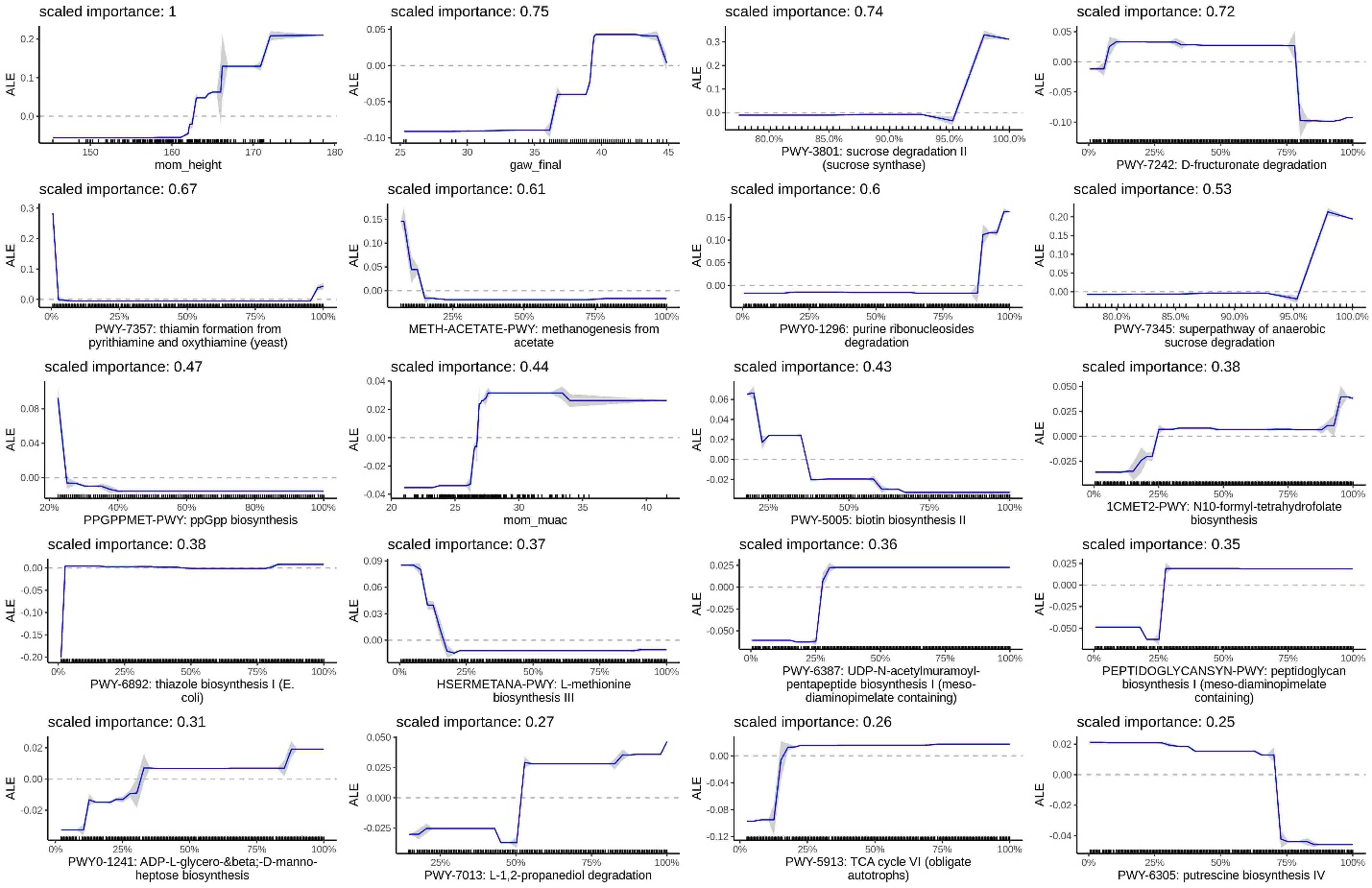


**Supplementary Figure 7. Relationships between infant birth weight in kg, epidemiologic variables, and maternal gut microbiome metabolic pathway relative abundance.** The top 20 predictors of infant birth weight by variable importance score are shown. For microbiome abundances, the x-axis represents the percentile of the abundance distribution. Epidemiologic and microbiome diversity variables are on the original scale. Tick marks on the x-axis are a rug plot of individual feature abundance percentiles. ALEs were generated using the *ALEplot* package and were plotted using *ggplot2*. Standard deviations (sd) were calculated per increment in microbiome feature and were used to calculate and plot increment-wise 95% confidence intervals as the average change in the outcome ±1.96(sd/sqrt(n)), where n is the number of observed feature values, and sd is the standard deviation of the change in the outcome variable in an interval. gaw_final, gestational age; mom_height, maternal height in centimeters; mom_muac, maternal mid-upper arm circumference in millimeters; pct_human, percent human reads; pct_unknown, percent unknown reads.


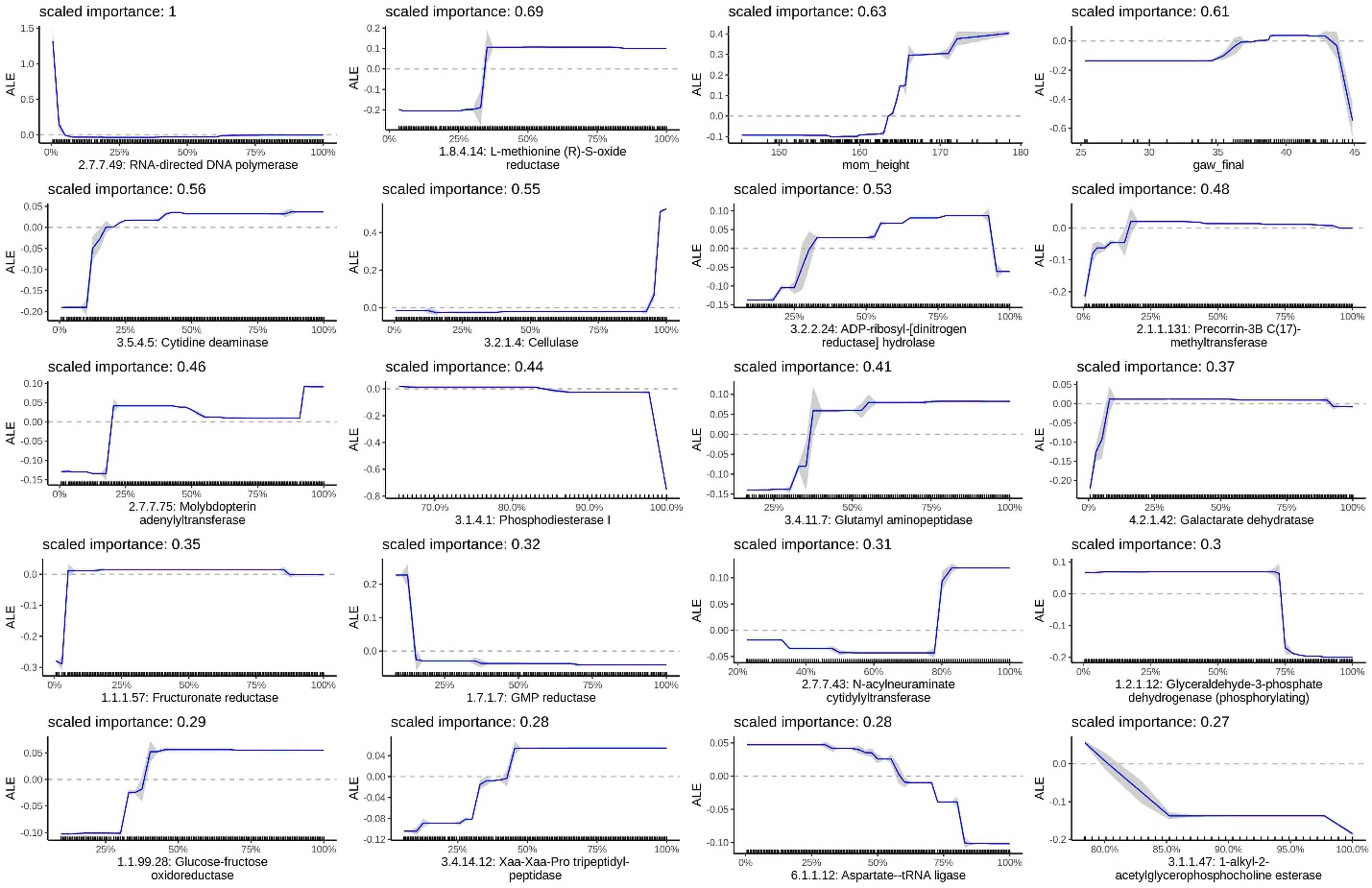


**Supplementary Figure 8.** **Relationships between infant WAZ at 1-month, epidemiologic variables, and maternal gut microbiome Enzyme Commission Categories (EC) relative abundance.** The top 20 predictors of infant WAZ at 1-month by variable importance score are shown. For microbiome abundances, the x-axis represents the percentile of the abundance distribution. Epidemiologic and microbiome diversity variables are on the original scale. Tick marks on the x-axis are a rug plot of individual feature abundance percentiles. ALEs were generated using the *ALEplot* package and were plotted using *ggplot2*. Standard deviations (sd) were calculated per increment in microbiome feature and were used to calculate and plot increment-wise 95% confidence intervals as the average change in the outcome ±1.96(sd/sqrt(n)), where n is the number of observed feature values, and sd is the standard deviation of the change in the outcome variable in an interval. gaw_final, gestational age; mom_height, maternal height in centimeters; mom_muac, maternal mid-upper arm circumference in millimeters; pct_human, percent human reads; pct_unknown, percent unknown reads.


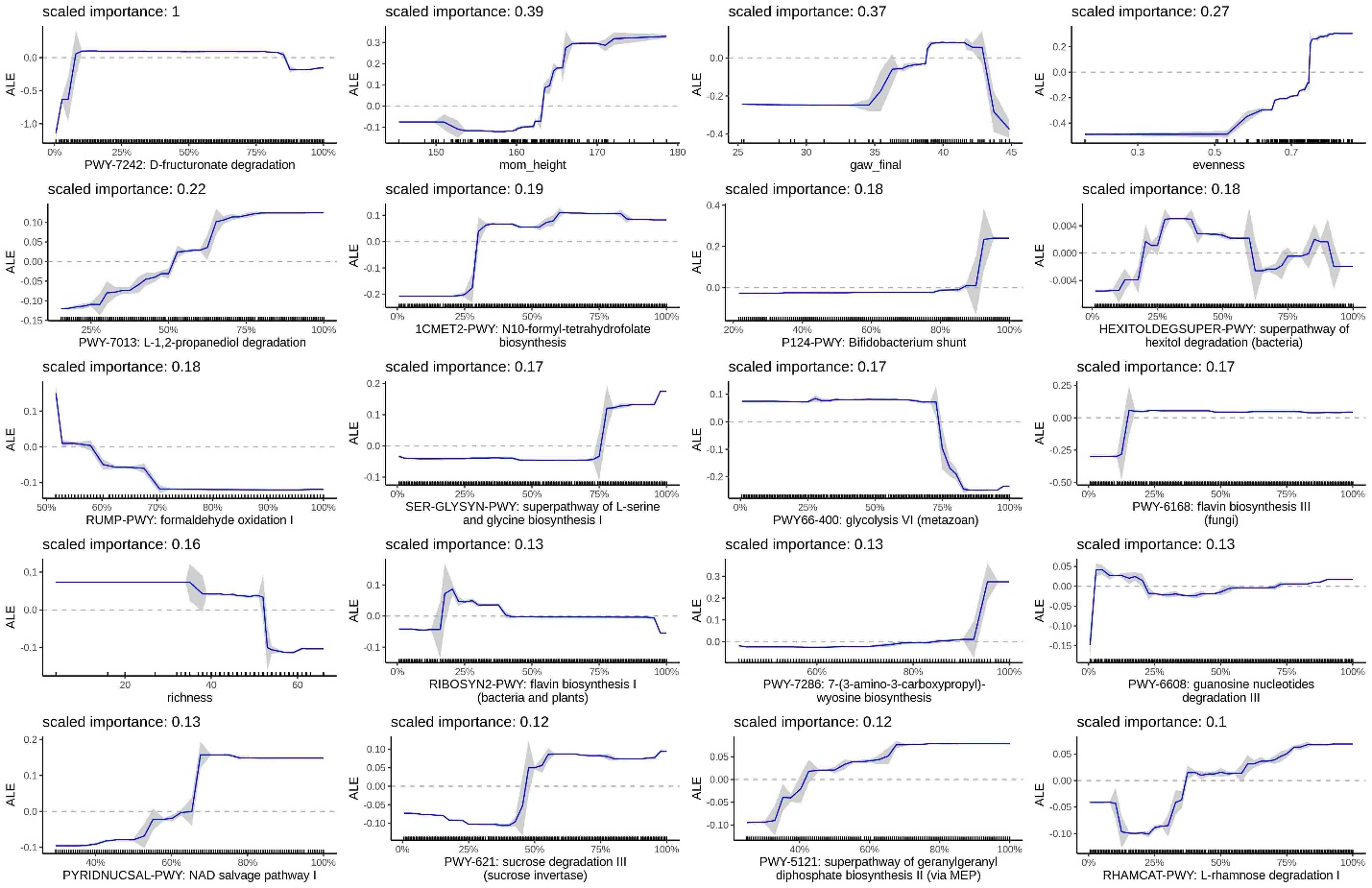


**Supplementary Figure 9.** **Relationships between infant WAZ at 1-month, epidemiologic variables, and maternal gut microbiome metabolic pathway relative abundance.** The top 20 predictors of infant WAZ at 1-month by variable importance score are shown. For microbiome abundances, the x-axis represents the percentile of the abundance distribution. Epidemiologic and microbiome diversity variables are on the original scale. Tick marks on the x-axis are a rug plot of individual feature abundance percentiles. ALEs were generated using the *ALEplot* package and were plotted using *ggplot2*. Standard deviations (sd) were calculated per increment in microbiome feature and were used to calculate and plot increment-wise 95% confidence intervals as the average change in the outcome ±1.96(sd/sqrt(n)), where n is the number of observed feature values, and sd is the standard deviation of the change in the outcome variable in an interval. gaw_final, gestational age; mom_height, maternal height in centimeters; mom_muac, maternal mid-upper arm circumference in millimeters; pct_human, percent human reads; pct_unknown, percent unknown reads.


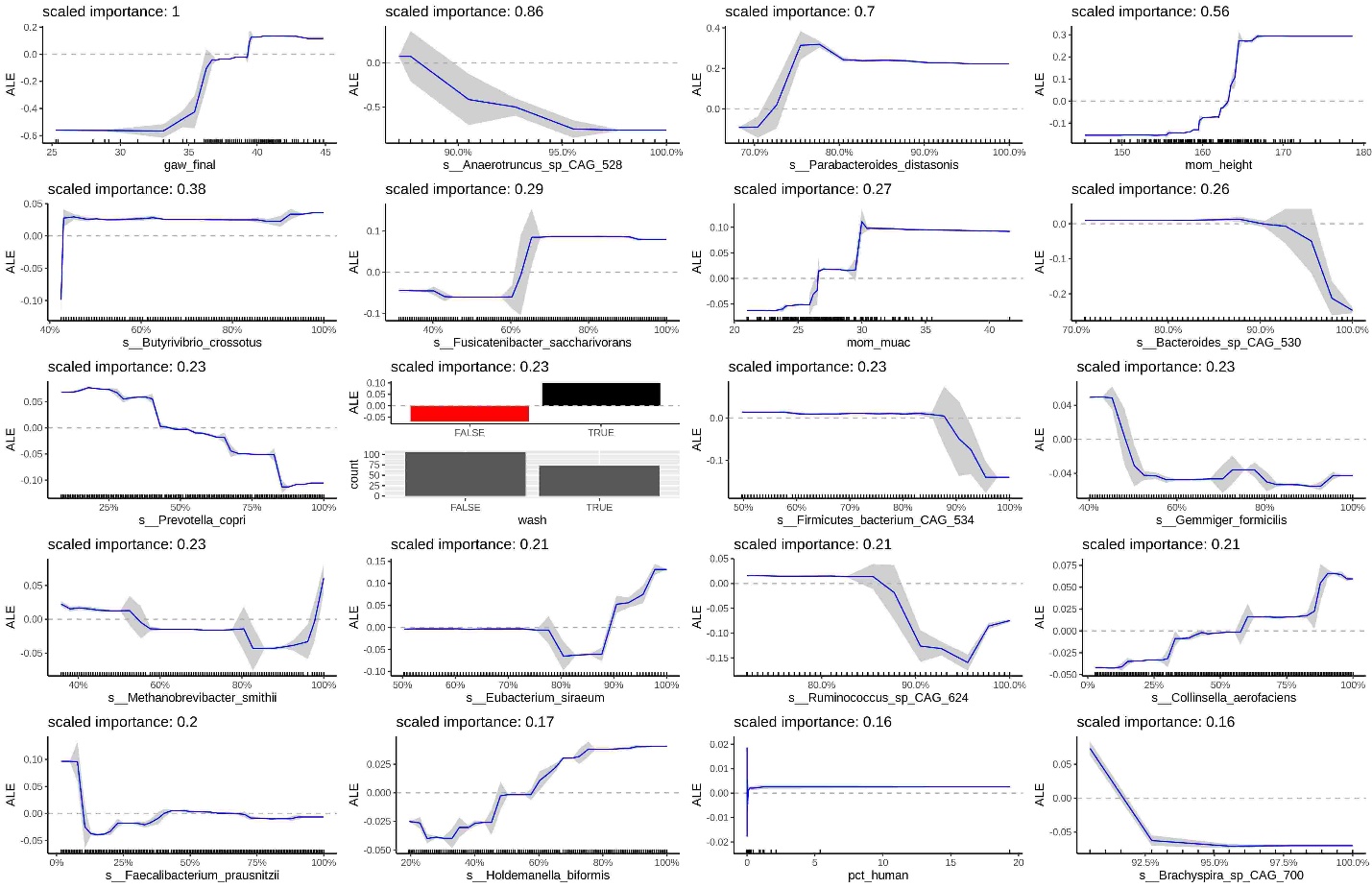


**Supplementary Figure 10.** **Relationships between infant LAZ at 1mo, epidemiologic variables, and maternal gut microbiome species relative abundance.** The top 20 predictors of infant LAZ at 1-month by variable importance score are shown. For microbiome abundances, the x-axis represents the percentile of the abundance distribution. Epidemiologic and microbiome diversity variables are on the original scale. Tick marks on the x-axis are a rug plot of individual feature abundance percentiles. ALEs were generated using the *ALEplot* package and were plotted using *ggplot2*. Standard deviations (sd) were calculated per increment in microbiome feature and were used to calculate and plot increment-wise 95% confidence intervals as the average change in the outcome ±1.96(sd/sqrt(n)), where n is the number of observed feature values, and sd is the standard deviation of the change in the outcome variable in an interval. gaw_final, gestational age; mom_height, maternal height in centimeters; mom_muac, maternal mid-upper arm circumference in millimeters; pct_human, percent human reads; pct_unknown, percent unknown reads.


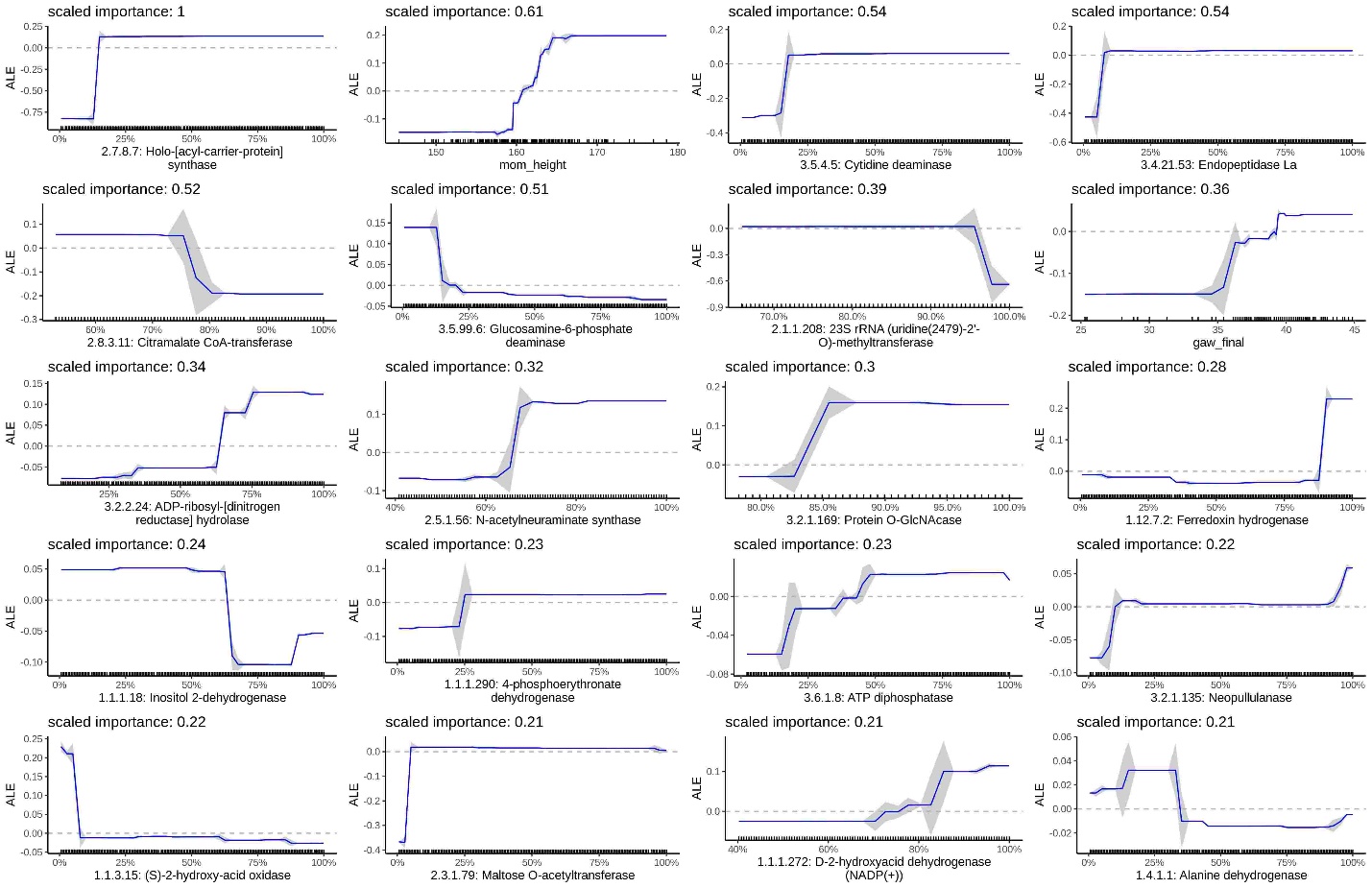


**Supplementary Figure 11.** **Relationships between infant LAZ at 1mo, epidemiologic variables, and maternal gut microbiome Enzyme Commission Categories (EC) relative abundance.** The top 20 predictors of LAZ at 1-month by variable importance score are shown. For microbiome abundances, the x-axis represents the percentile of the abundance distribution. Epidemiologic and microbiome diversity variables are on the original scale. Tick marks on the x-axis are a rug plot of individual feature abundance percentiles. ALEs were generated using the *ALEplot* package and were plotted using *ggplot2*. Standard deviations (sd) were calculated per increment in microbiome feature and were used to calculate and plot increment-wise 95% confidence intervals as the average change in the outcome ±1.96(sd/sqrt(n)), where n is the number of observed feature values, and sd is the standard deviation of the change in the outcome variable in an interval. gaw_final, gestational age; mom_height, maternal height in centimeters; mom_muac, maternal mid-upper arm circumference in millimeters; pct_human, percent human reads; pct_unknown, percent unknown reads.


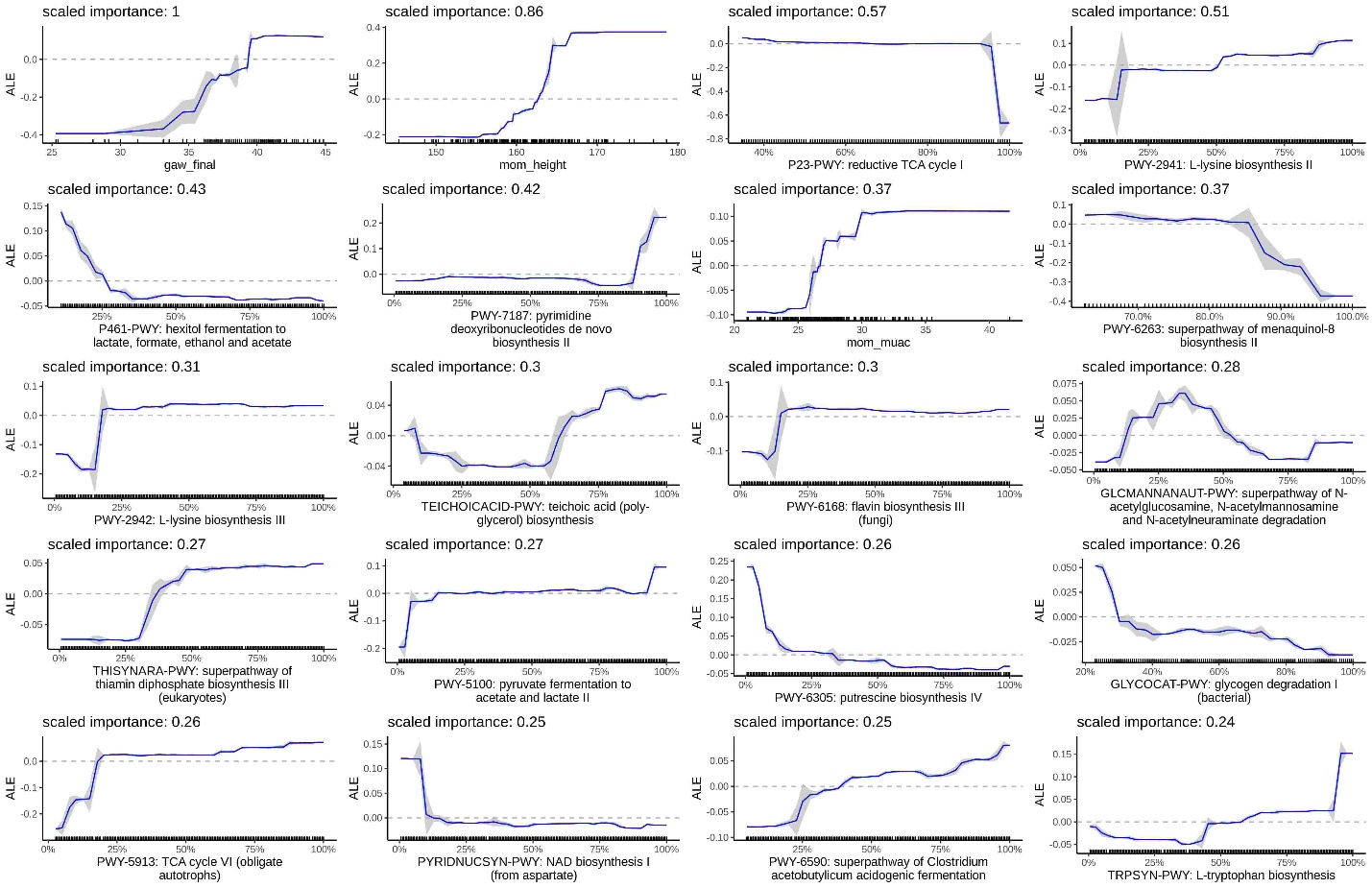


**Supplementary Figure 12.** **Relationships between infant LAZ at 1mo, epidemiologic variables, and maternal gut microbiome metabolic pathway relative abundance.** The top 20 predictors of infant LAZ at 1-month by variable importance score are shown. For microbiome abundances, the x-axis represents the percentile of the abundance distribution. Epidemiologic and microbiome diversity variables are on the original scale. Tick marks on the x-axis are a rug plot of individual feature abundance percentiles. ALEs were generated using the *ALEplot* package and were plotted using *ggplot2*. Standard deviations (sd) were calculated per increment in microbiome feature and were used to calculate and plot increment-wise 95% confidence intervals as the average change in the outcome ±1.96(sd/sqrt(n)), where n is the number of observed feature values, and sd is the standard deviation of the change in the outcome variable in an interval. gaw_final, gestational age; mom_height, maternal height in centimeters; mom_muac, maternal mid-upper arm circumference in millimeters; pct_human, percent human reads; pct_unknown, percent unknown reads.


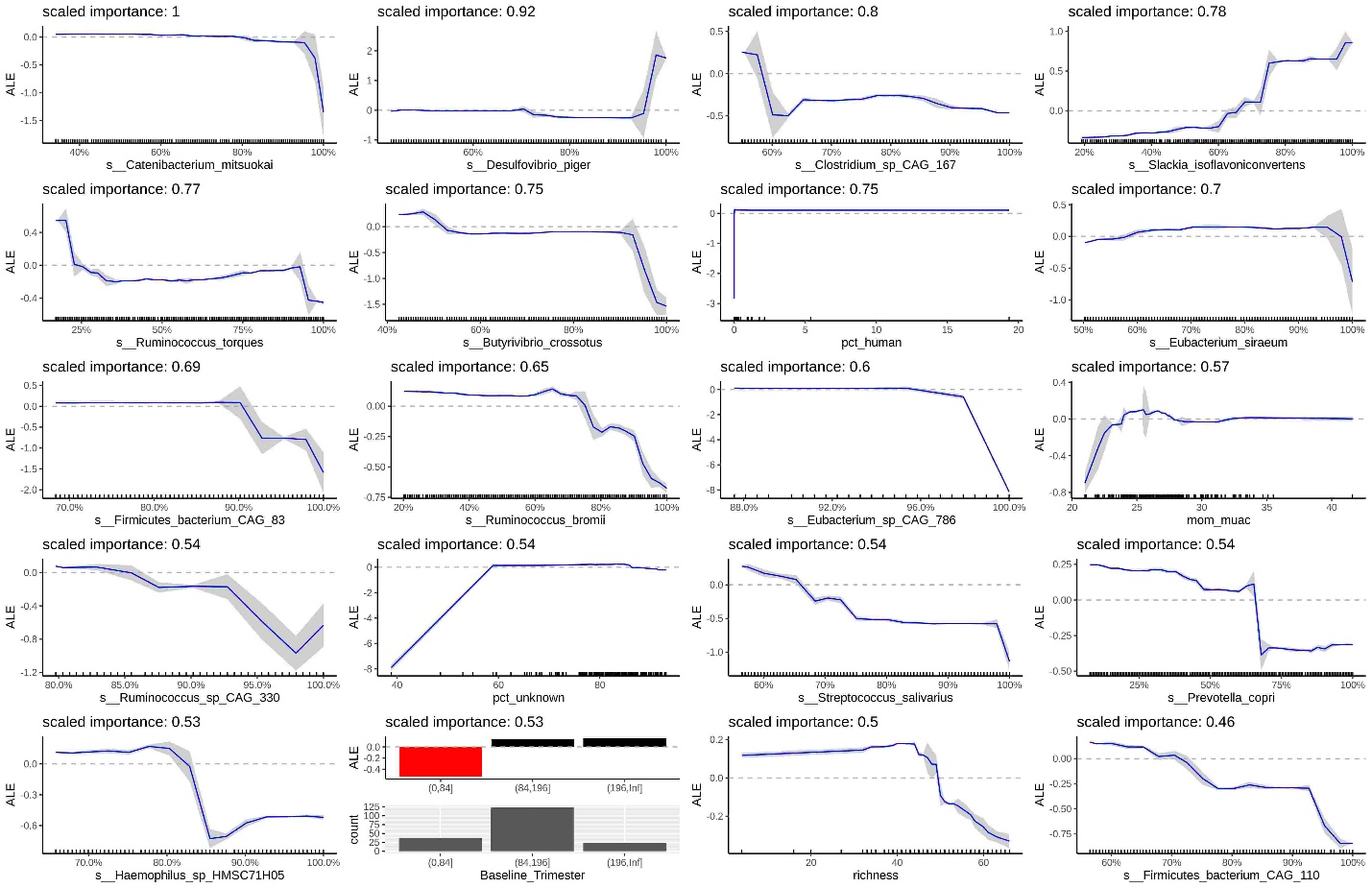


**Supplementary Figure 13.** **Relationships between infant gestational age in weeks, epidemiologic variables, and maternal gut microbiome species relative abundance.** The top 20 predictors of infant gestational age by variable importance score are shown. For microbiome abundances, the x-axis represents the percentile of the abundance distribution. Epidemiologic and microbiome diversity variables are on the original scale. Tick marks on the x-axis are a rug plot of individual feature abundance percentiles. ALEs were generated using the *ALEplot* package and were plotted using *ggplot2*. Standard deviations (sd) were calculated per increment in microbiome feature and were used to calculate and plot increment-wise 95% confidence intervals as the average change in the outcome ±1.96(sd/sqrt(n)), where n is the number of observed feature values, and sd is the standard deviation of the change in the outcome variable in an interval. gaw_final, gestational age; mom_height, maternal height in centimeters; mom_muac, maternal mid-upper arm circumference in millimeters; pct_human, percent human reads; pct_unknown, percent unknown reads.


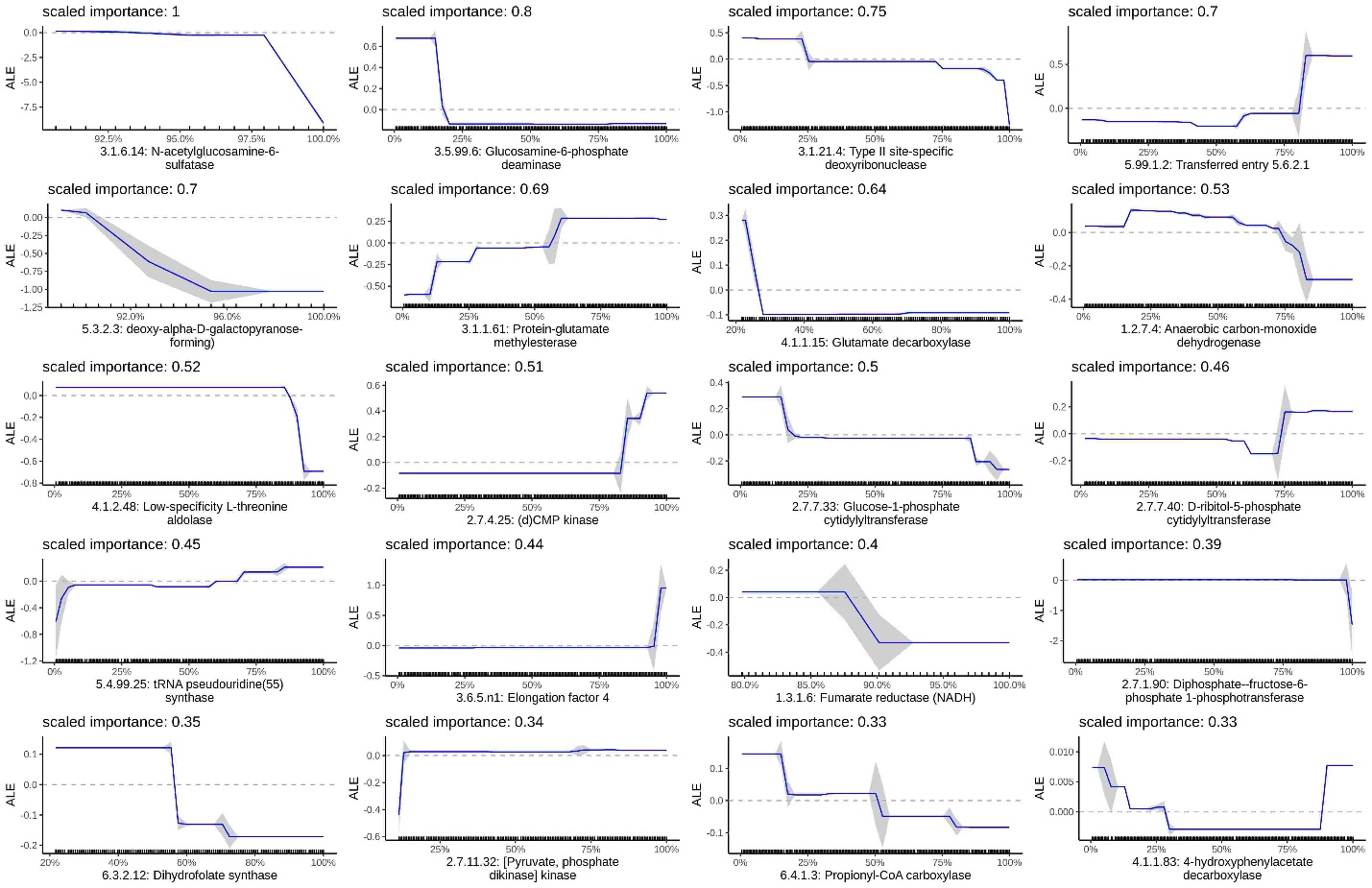


**Supplementary Figure 14. Relationships between infant gestational age in weeks, epidemiologic variables, and maternal gut microbiome Enzyme Commission Categories (EC) relative abundance.** The top 20 predictors of infant gestational age by variable importance score are shown. For microbiome abundances, the x-axis represents the percentile of the abundance distribution. Epidemiologic and microbiome diversity variables are on the original scale. Tick marks on the x-axis are a rug plot of individual feature abundance percentiles. ALEs were generated using the *ALEplot* package and were plotted using *ggplot2*. Standard deviations (sd) were calculated per increment in microbiome feature and were used to calculate and plot increment-wise 95% confidence intervals as the average change in the outcome ±1.96(sd/sqrt(n)), where n is the number of observed feature values, and sd is the standard deviation of the change in the outcome variable in an interval. gaw_final, gestational age; mom_height, maternal height in centimeters; mom_muac, maternal mid-upper arm circumference in millimeters; pct_human, percent human reads; pct_unknown, percent unknown reads.


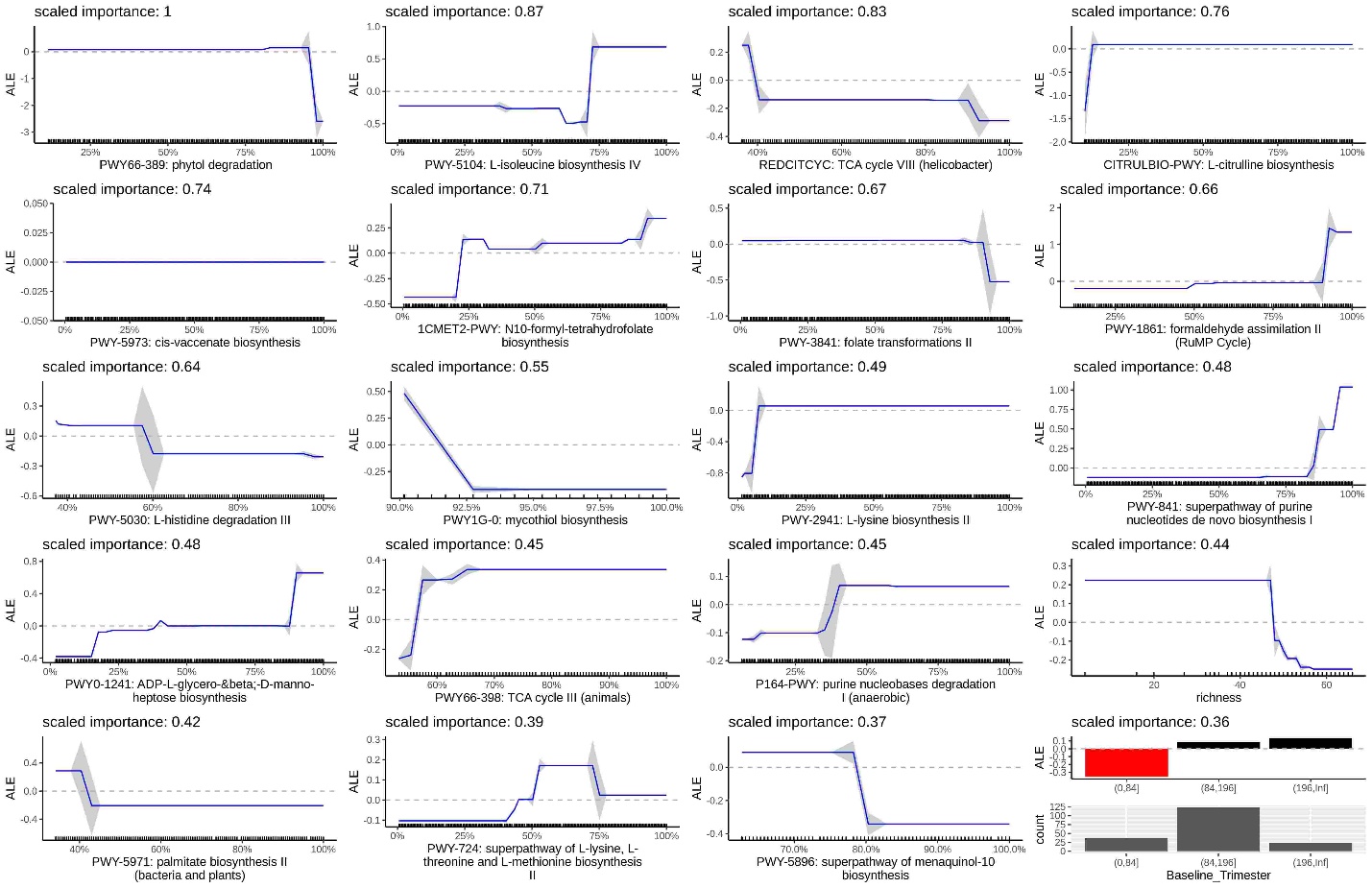


**Supplementary Figure 15.** **Relationships between infant gestational age in weeks, epidemiologic variables, and maternal gut microbiome metabolic pathway relative abundance.** The top 20 predictors of infant gestational age by variable importance score are shown. For microbiome abundances, the x-axis represents the percentile of the abundance distribution. Epidemiologic and microbiome diversity variables are on the original scale. Tick marks on the x-axis are a rug plot of individual feature abundance percentiles. ALEs were generated using the *ALEplot* package and were plotted using *ggplot2*. Standard deviations (sd) were calculated per increment in microbiome feature and were used to calculate and plot increment-wise 95% confidence intervals as the average change in the outcome ±1.96(sd/sqrt(n)), where n is the number of observed feature values, and sd is the standard deviation of the change in the outcome variable in an interval. gaw_final, gestational age; mom_height, maternal height in centimeters; mom_muac, maternal mid-upper arm circumference in millimeters; pct_human, percent human reads; pct_unknown, percent unknown reads.
