# Supplementary material for: Maternal fecal microbiome predicts gestational age, birth weight and neonatal growth in rural Zimbabwe
Source: eBioMedicine. 2021 Jun 15;68:103421. doi: 10.1016/j.ebiom.2021.103421 (PMC8217692; doi:10.1016/j.ebiom.2021.103421)
Supplement: Supplementary file 2 [file mmc2.docx]

**Supplementary Table 1. Grid search hyperparameters for all XGBoost models**

**Supplementary Table 2. Diet consumption on a normal day among the sub-sample of mothers included in the microbiome sub-study**

**Supplementary Table 3. Influence of baseline variables on maternal fecal microbiota during and after pregnancy**

**Supplementary Table 4. Epidemiologic variables associated with infant birthweight, LAZ and WAZ at 1mo of age in multivariable linear regression models.** SE, standard error; Kg, kilograms; cm, centimeters, WASH, water, sanitation, and hygiene intervention arm.

**Supplementary Table 5. XGBoost performance parameters for maternal microbiome relative abundance and gestational age, fetal or neonatal growth.** *1, microbiome relative abundance + diversity metrics + epidemiologic variables; 2, microbiome relative abundance + diversity metrics; 3, microbiome relative abundance. **R-squared between observed and model predicted outcome values.

**Supplementary Figure 1. Distributions of (log-transformed) percent human DNA and percent non-annotatable sequencing reads from maternal microbiome datasets.**

**Supplementary Figure 2. Principal coordinate analysis based on Bray-Curtis dissimilarity index for maternal fecal microbiomes by maternal HIV status, specimen collection visit, season of specimen collection, or randomized WASH arm.** 95% confidence ellipses are provided.

**Supplementary Figure 3. Principal coordinate analysis based on Bray-Curtis dissimilarity index for maternal fecal microbiomes by trimester of Pregnancy.** 95% confidence ellipses are provided.

**Supplementary Figure 4. The distribution of prevalent (present in ≥ 5% of SHINE mothers) and abundant (present at ≥ 1% relative abundance) taxa in the gestational visit (top) and in the one-month post-partum (bottom) visit, as defined by MetaPhlAn3.** Blue outlined boxes are taxa that are present at only one visit.

**Supplementary Figure 5. Relationship between α-diversity measures and select infant growth outcomes. Relationship between α-diversity measures and select infant growth outcomes.** A single dataset with very low (> 4 s.d.) α-diversity measures was removed from the analyses.

**Supplementary Figure 6. Relationships between infant birth weight in kg, maternal characteristics, and maternal gut microbiome Enzyme Commission Categories (EC) relative abundance.** The top 20 predictors of infant birth weight by variable importance score are shown. For microbiome abundances, the x-axis represents the percentile of the abundance distribution. Epidemiologic and microbiome diversity variables are on the original scale. Tick marks on the x-axis are a rug plot of individual feature abundance percentiles. ALEs were generated using the *ALEplot* package and were plotted using *ggplot2*. Standard deviations (sd) were calculated per increment in microbiome feature and were used to calculate and plot increment-wise 95% confidence intervals as the average change in the outcome ±1.96(sd/sqrt(n)), where n is the number of observed feature values, and sd is the standard deviation of the change in the outcome variable in an interval. gaw_final, gestational age; mom_height, maternal height in centimeters; mom_muac, maternal mid-upper arm circumference in millimeters; pct_human, percent human reads; pct_unknown, percent unknown reads.

**Supplementary Figure 7. Relationships between infant birth weight in kg, epidemiologic variables, and maternal gut microbiome metabolic pathway relative abundance.** The top 20 predictors of infant birth weight by variable importance score are shown. For microbiome abundances, the x-axis represents the percentile of the abundance distribution. Epidemiologic and microbiome diversity variables are on the original scale. Tick marks on the x-axis are a rug plot of individual feature abundance percentiles. ALEs were generated using the *ALEplot* package and were plotted using *ggplot2*. Standard deviations (sd) were calculated per increment in microbiome feature and were used to calculate and plot increment-wise 95% confidence intervals as the average change in the outcome ±1.96(sd/sqrt(n)), where n is the number of observed feature values, and sd is the standard deviation of the change in the outcome variable in an interval. gaw_final, gestational age; mom_height, maternal height in centimeters; mom_muac, maternal mid-upper arm circumference in millimeters; pct_human, percent human reads; pct_unknown, percent unknown reads.

**Supplementary Figure 8.** **Relationships between infant WAZ at 1-month, epidemiologic variables, and maternal gut microbiome Enzyme Commission Categories (EC) relative abundance.** The top 20 predictors of infant WAZ at 1-month by variable importance score are shown. For microbiome abundances, the x-axis represents the percentile of the abundance distribution. Epidemiologic and microbiome diversity variables are on the original scale. Tick marks on the x-axis are a rug plot of individual feature abundance percentiles. ALEs were generated using the *ALEplot* package and were plotted using *ggplot2*. Standard deviations (sd) were calculated per increment in microbiome feature and were used to calculate and plot increment-wise 95% confidence intervals as the average change in the outcome ±1.96(sd/sqrt(n)), where n is the number of observed feature values, and sd is the standard deviation of the change in the outcome variable in an interval. gaw_final, gestational age; mom_height, maternal height in centimeters; mom_muac, maternal mid-upper arm circumference in millimeters; pct_human, percent human reads; pct_unknown, percent unknown reads.

**Supplementary Figure 9.** **Relationships between infant WAZ at 1-month, epidemiologic variables, and maternal gut microbiome metabolic pathway relative abundance.** The top 20 predictors of infant WAZ at 1-month by variable importance score are shown. For microbiome abundances, the x-axis represents the percentile of the abundance distribution. Epidemiologic and microbiome diversity variables are on the original scale. Tick marks on the x-axis are a rug plot of individual feature abundance percentiles. ALEs were generated using the *ALEplot* package and were plotted using *ggplot2*. Standard deviations (sd) were calculated per increment in microbiome feature and were used to calculate and plot increment-wise 95% confidence intervals as the average change in the outcome ±1.96(sd/sqrt(n)), where n is the number of observed feature values, and sd is the standard deviation of the change in the outcome variable in an interval. gaw_final, gestational age; mom_height, maternal height in centimeters; mom_muac, maternal mid-upper arm circumference in millimeters; pct_human, percent human reads; pct_unknown, percent unknown reads.

**Supplementary Figure 10.** **Relationships between infant LAZ at 1mo, epidemiologic variables, and maternal gut microbiome species relative abundance.** The top 20 predictors of infant LAZ at 1-month by variable importance score are shown. For microbiome abundances, the x-axis represents the percentile of the abundance distribution. Epidemiologic and microbiome diversity variables are on the original scale. Tick marks on the x-axis are a rug plot of individual feature abundance percentiles. ALEs were generated using the *ALEplot* package and were plotted using *ggplot2*. Standard deviations (sd) were calculated per increment in microbiome feature and were used to calculate and plot increment-wise 95% confidence intervals as the average change in the outcome ±1.96(sd/sqrt(n)), where n is the number of observed feature values, and sd is the standard deviation of the change in the outcome variable in an interval. gaw_final, gestational age; mom_height, maternal height in centimeters; mom_muac, maternal mid-upper arm circumference in millimeters; pct_human, percent human reads; pct_unknown, percent unknown reads.

**Supplementary Figure 11.** **Relationships between infant LAZ at 1mo, epidemiologic variables, and maternal gut microbiome Enzyme Commission Categories (EC) relative abundance.** The top 20 predictors of LAZ at 1-month by variable importance score are shown. For microbiome abundances, the x-axis represents the percentile of the abundance distribution. Epidemiologic and microbiome diversity variables are on the original scale. Tick marks on the x-axis are a rug plot of individual feature abundance percentiles. ALEs were generated using the *ALEplot* package and were plotted using *ggplot2*. Standard deviations (sd) were calculated per increment in microbiome feature and were used to calculate and plot increment-wise 95% confidence intervals as the average change in the outcome ±1.96(sd/sqrt(n)), where n is the number of observed feature values, and sd is the standard deviation of the change in the outcome variable in an interval. gaw_final, gestational age; mom_height, maternal height in centimeters; mom_muac, maternal mid-upper arm circumference in millimeters; pct_human, percent human reads; pct_unknown, percent unknown reads.

**Supplementary Figure 12.** **Relationships between infant LAZ at 1mo, epidemiologic variables, and maternal gut microbiome metabolic pathway relative abundance.** The top 20 predictors of infant LAZ at 1-month by variable importance score are shown. For microbiome abundances, the x-axis represents the percentile of the abundance distribution. Epidemiologic and microbiome diversity variables are on the original scale. Tick marks on the x-axis are a rug plot of individual feature abundance percentiles. ALEs were generated using the *ALEplot* package and were plotted using *ggplot2*. Standard deviations (sd) were calculated per increment in microbiome feature and were used to calculate and plot increment-wise 95% confidence intervals as the average change in the outcome ±1.96(sd/sqrt(n)), where n is the number of observed feature values, and sd is the standard deviation of the change in the outcome variable in an interval. gaw_final, gestational age; mom_height, maternal height in centimeters; mom_muac, maternal mid-upper arm circumference in millimeters; pct_human, percent human reads; pct_unknown, percent unknown reads.

**Supplementary Figure 13.** **Relationships between infant gestational age in weeks, epidemiologic variables, and maternal gut microbiome species relative abundance.** The top 20 predictors of infant gestational age by variable importance score are shown. For microbiome abundances, the x-axis represents the percentile of the abundance distribution. Epidemiologic and microbiome diversity variables are on the original scale. Tick marks on the x-axis are a rug plot of individual feature abundance percentiles. ALEs were generated using the *ALEplot* package and were plotted using *ggplot2*. Standard deviations (sd) were calculated per increment in microbiome feature and were used to calculate and plot increment-wise 95% confidence intervals as the average change in the outcome ±1.96(sd/sqrt(n)), where n is the number of observed feature values, and sd is the standard deviation of the change in the outcome variable in an interval. gaw_final, gestational age; mom_height, maternal height in centimeters; mom_muac, maternal mid-upper arm circumference in millimeters; pct_human, percent human reads; pct_unknown, percent unknown reads.

**Supplementary Figure 14. Relationships between infant gestational age in weeks, epidemiologic variables, and maternal gut microbiome Enzyme Commission Categories (EC) relative abundance.** The top 20 predictors of infant gestational age by variable importance score are shown. For microbiome abundances, the x-axis represents the percentile of the abundance distribution. Epidemiologic and microbiome diversity variables are on the original scale. Tick marks on the x-axis are a rug plot of individual feature abundance percentiles. ALEs were generated using the *ALEplot* package and were plotted using *ggplot2*. Standard deviations (sd) were calculated per increment in microbiome feature and were used to calculate and plot increment-wise 95% confidence intervals as the average change in the outcome ±1.96(sd/sqrt(n)), where n is the number of observed feature values, and sd is the standard deviation of the change in the outcome variable in an interval. gaw_final, gestational age; mom_height, maternal height in centimeters; mom_muac, maternal mid-upper arm circumference in millimeters; pct_human, percent human reads; pct_unknown, percent unknown reads.

**Supplementary Figure 15.** **Relationships between infant gestational age in weeks, epidemiologic variables, and maternal gut microbiome metabolic pathway relative abundance.** The top 20 predictors of infant gestational age by variable importance score are shown. For microbiome abundances, the x-axis represents the percentile of the abundance distribution. Epidemiologic and microbiome diversity variables are on the original scale. Tick marks on the x-axis are a rug plot of individual feature abundance percentiles. ALEs were generated using the *ALEplot* package and were plotted using *ggplot2*. Standard deviations (sd) were calculated per increment in microbiome feature and were used to calculate and plot increment-wise 95% confidence intervals as the average change in the outcome ±1.96(sd/sqrt(n)), where n is the number of observed feature values, and sd is the standard deviation of the change in the outcome variable in an interval. gaw_final, gestational age; mom_height, maternal height in centimeters; mom_muac, maternal mid-upper arm circumference in millimeters; pct_human, percent human reads; pct_unknown, percent unknown reads.
