# Supplementary material for: Maternal fecal microbiome predicts gestational age, birth weight and neonatal growth in rural Zimbabwe
Source: eBioMedicine. 2021 Jun 15;68:103421. doi: 10.1016/j.ebiom.2021.103421 (PMC8217692; doi:10.1016/j.ebiom.2021.103421)
Supplement: Supplementary file 3 [file mmc3.pdf]

Consent for Acknowledgments

Manuscript Title: **Maternal fecal microbiome predicts gestational age, birth weight and neonatal growth in rural Zimbabwe.**
Corresponding author: **Amee R Manges**

Article type: **Original Research**

<u>Person or entity being acknowledged to fill in items below:</u>

I have seen a version of the manuscript to be submitted/published and I hereby give my consent for my name (or name of entity) to be included in the acknowledgement section in the above-named manuscript for consideration of publication in the *EBioMedicine.* I understand that this signed form will be submitted to the journal with the manuscript as evidence of my consent.

I understand that this manuscript may be published in the *EBioMedicine* and in products derived from the journals. As a result, I understand that the material may be seen by the general public. I understand that I may revoke consent at any time before publication, but once the information has been published revocation of the consent is no longer possible. I understand that I will derive no financial benefit from publication of this paper.

Phillipa Rambanepasi
Name of Person (print name) and/or Entity (print entity name and name of person signing)

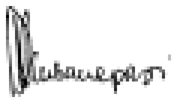

____

Signature (or signature of the person giving consent on behalf of the person)

30 April 2021
Date

Only complete this section if you are <u>not</u> the person. What is your relationship? (The person giving consent should be a substitute decision maker or legal guardian or should hold power of attorney for the person or entity.)

____

Why is the person not able to give consent? (e.g., is the person a minor, incapacitated, or deceased?)

Consent for Acknowledgments

Manuscript Title: **Maternal fecal microbiome predicts gestational age, birth weight and neonatal growth in rural Zimbabwe.**

Corresponding author: **Amee R Manges**

Article type: **Original Research**

Person or entity being acknowledged to fill in items below:

I have seen a version of the manuscript to be submitted/published and I hereby give my consent for my name (or name of entity) to be included in the acknowledgement section in the above-named manuscript for consideration of publication in the *EBioMedicine.* I understand that this signed form will be submitted to the journal with the manuscript as evidence of my consent.

I understand that this manuscript may be published in the *EBioMedicine* and in products derived from the journals. As a result, I understand that the material may be seen by the general public. I understand that I may revoke consent at any time before publication, but once the information has been published revocation of the consent is no longer possible. I understand that I will derive no financial benefit from publication of this paper.

Virginia Sauramba

Name of Person (print name) and/or Entity (print entity name and name of person signing)

____

Signature (or signature of the person giving consent on behalf of the person)

Date : 29 April 2021

Only complete this section if you are not the person. What is your relationship? (The person giving consent should be a substitute decision maker or legal guardian or should hold power of attorney for the person or entity.)

____

Why is the person not able to give consent? (e.g., is the person a minor, incapacitated, or deceased?)
